# Increasing the use of medical rehabilitation by children and adolescents with migrant background through a multimodal information campaign: protocol of a trend study and accompanying process evaluation (MiMi-Reha-Kids, DRKS00019090)

**DOI:** 10.3389/fpubh.2023.1089685

**Published:** 2023-07-14

**Authors:** Hannes Banaschak, Flaminia Bartolini, Ramazan Salman, Matthias Bethge

**Affiliations:** ^1^Institute for Social Medicine and Epidemiology, University of Lübeck, Lübeck, Germany; ^2^Ethno-Medical Center, Hannover, Germany

**Keywords:** complex intervention, community, rehabilitation, utilization, migrant, health inequalities, children, health literacy

## Abstract

**Background:**

Chronic illnesses can have an unfavorable impact on the participation opportunities of children and adolescents. The German health care system offers medical rehabilitation in order to prevent negative effects, however, migrant children and adolescents make use of this option less frequently than their peers without a migrant background. A multimodal information campaign was developed to increase the use of medical rehabilitation by children and adolescents with a migrant background, and to reduce disparities in health care.

**Methods:**

The process evaluation will examine the implementation of a multimodal information campaign intended to increase the use of medical rehabilitation by migrant children and adolescents. The information campaign follows a low-threshold participatory approach. In a first step, persons from different migrant communities in Berlin and Hamburg are trained to become transcultural health mediators. These mediators then share their knowledge about chronic illnesses and medical rehabilitation with other families at information events held in their native language. The transcultural mediators also support migrant families in applying for medical rehabilitation. The effectiveness of the intervention will be tested by a trend study with repeated cross-sectional surveys. For this purpose, all families in the project regions of Berlin and Hamburg whose child has received medical rehabilitation are surveyed annually in order to be able to map changes in the proportions of children and adolescents with a migrant background over the course of the project.

**Discussion:**

The study protocol describes a complex intervention to increase the use of medical rehabilitation by migrant children and adolescents, and the accompanying process evaluation and trend study. The intervention is intended to contribute to reducing health inequalities in Germany.

**Conclusion:**

The study described in this protocol will provide extensive data on the multimodal information campaign and can thus help organizations and institutions adapt or further develop similar measures for other regions.

**Clinical trial registration:**

German Clinical Trials Register (DRKS00019090).

## 1. Introduction

Worldwide, the number of international migrants has grown significantly over the past 50 years. While in 1970 about 84 million people, or 2.3% of the world’s population at that time, lived in a country other than the one of their birth, in 2020 there were about 280 million people, or 3.6% of the world’s population (1). The reasons for migration movements are complex. In addition to civil unrest, war events, and natural disasters, economic and political conditions can be a major factor. Migration movements raise different challenges for countries of origin as well as destination countries. For example, while origin countries struggle with a loss of knowledge and skills, destination countries are required to create conditions that enable inclusion and inclusive transformation of structures in the different sectors of the social system. This includes the domain of health care, which must respond to citizens with different biographical experiences and resulting expectations and needs. In this paper, we describe a multimodal information campaign and associated evaluation that is designed to improve health care for migrant children in Germany.

### 1.1. Germany as a country of immigration

German society today is characterized by a variety of different ethnic, cultural and social backgrounds of its citizens. There have been various waves of migration since the foundation of the Federal Republic of Germany and the German Democratic Republic in 1949. In the first years after the end of World War II, it was mainly people from the former eastern territories (i.e., territories of today’s Poland and Russia) who moved to the Federal Republic of Germany, and they were followed in the 1950s by migrant workers from countries such as Italy, Spain, Greece, Turkey and Portugal. In addition to a steady influx of late re-settlers from states of the former Soviet Union, people fleeing their countries due to wars and civil wars (e.g., the Bosnian War of 1992–1995) found a new home in Germany. Although numerically inferior to migration to Western Germany, the German Democratic Republic also experienced the arrival of migrant workers from countries, such as Vietnam, Mozambique and Cuba. More recently, free movement for citizens of EU member states, which has been guaranteed by the European Union since 1993, has made it easier for people with diverse migration biographies to live in Germany. According to OECD data, Germany is nowadays the most common immigration destination after the USA (2). In 2019, around 26% of the total population in Germany had a migrant background (MB), i.e., their own or family migration history. In the under-18 age group, the proportion was around 39% (3).

### 1.2. Migrant health in Germany

Although the individual circumstances of children and adolescents with a MB can vary widely depending on family history, surveys show that children and adolescents with MB tend to be socioeconomically disadvantaged, and, for example, have a higher risk of poverty than their peers without a MB (4). Lower socioeconomic status is also associated with more frequent health problems and health-related risks in children and adolescents, as it is in adults (5, 6). This also applies to chronic diseases (e.g., obesity, attention deficit hyperactivity disorder), which are of particular importance because they can permanently jeopardize age-appropriate development and restrict the social participation of children and adolescents in the long term (7, 8). Chronic illnesses in children can also lead to psychosocial stress for close relatives such as parents and siblings (9, 10). Epidemiological studies estimate the proportion of chronically ill children in Germany to be about 16.2% (11). A migrant background in Germany is not a risk factor for a chronic disease in children and adolescents *per se*, however, there is a clear correlation between socioeconomic status and various disease patterns, such as obesity or mental disorders, which is why children and adolescents with MB are also more frequently affected by some diseases due to their comparatively low socioeconomic status (12).

### 1.3. Medical rehabilitation for children and adolescents

In general, children with chronic diseases in Germany are treated on an outpatient basis close to home by pediatricians or family doctors, in collaboration with other therapists (e.g., physiotherapists, child and adolescent psychotherapists). Outpatient treatment, however, may be not sufficient for some individuals. In these cases, the German social insurance system offers medical rehabilitation for children and adolescents (MRCA) (13). The legal basis for this is § 15a SGB VI. MRCA is intended to counteract the consequences of chronic illness, to enable schooling and vocational training without restrictions, and, in the long term, to prevent endangerment to, or the restriction of, gainful employment. In Germany, statutory health insurance and German Pension Insurance (GPI) are in principle equally responsible for medical rehabilitation. Despite this, the primary provider of MRCA services is the GPI. Every year, more than 32,000 requests for medical rehabilitation for children and adolescents are submitted to the German Pension Insurance. In Germany, medical rehabilitation for children and adolescents takes place almost exclusively in inpatient rehabilitation clinics. The most common reasons for using MRCA in 2018 were mental and behavioral disorders, followed by bronchial asthma, obesity and skin diseases such as neurodermatitis (14). Reliable data on the utilization of medical rehabilitation by children and adolescents with MB is not currently available, as the GPI administratively only records the nationality of rehabilitants. In terms of nationality, however, there is a clear underrepresentation of children and adolescents of non-German nationality (14). A recent study with adults shows that in addition to underutilization among persons without German nationality, underutilization by persons with a migration history can also be assumed (15). Possible reasons for this are utilization barriers such as insufficient language skills, a different understanding of illness, or insufficient knowledge of the German health care system (16).

### 1.4. Promoting change

The current care situation of children and adolescents with MB has encouraged the development and implementation of a complex intervention to increase the use of medical rehabilitation by children and adolescents with MB. We hypothesized that an increase in utilization could be achieved by increasing awareness of chronic conditions and the options for medical rehabilitation by offering low-threshold proactive information. Although there is already a variety of information on medical rehabilitation for children and adolescents, it is often only available in German. Moreover, there is also a question regarding the extent to which the current dissemination channels (e.g., via websites and counseling centers of official institutions) are suitable for reaching migrants in Germany. As the World Health Organization (WHO) has pointed out, the inclusion of migrants or migrant communities in the provision of information can be a suitable means of successful health promotion (17). Our project “Implementation and evaluation of a multilingual information campaign on rehabilitation for children and adolescents with a migrant background (MiMi-Reha-Kids)” follows this approach, in which migrants are actively involved in health education within the framework of a multimodal information campaign, and knowledge is consolidated in the migrant communities. The aim of the accompanying process evaluation and trend study is to clarify whether the information campaign increases access to and utilization of medical rehabilitation among children and adolescents with a migrant background.

## 2. Methods

### 2.1. Setting

**Figure 1:**
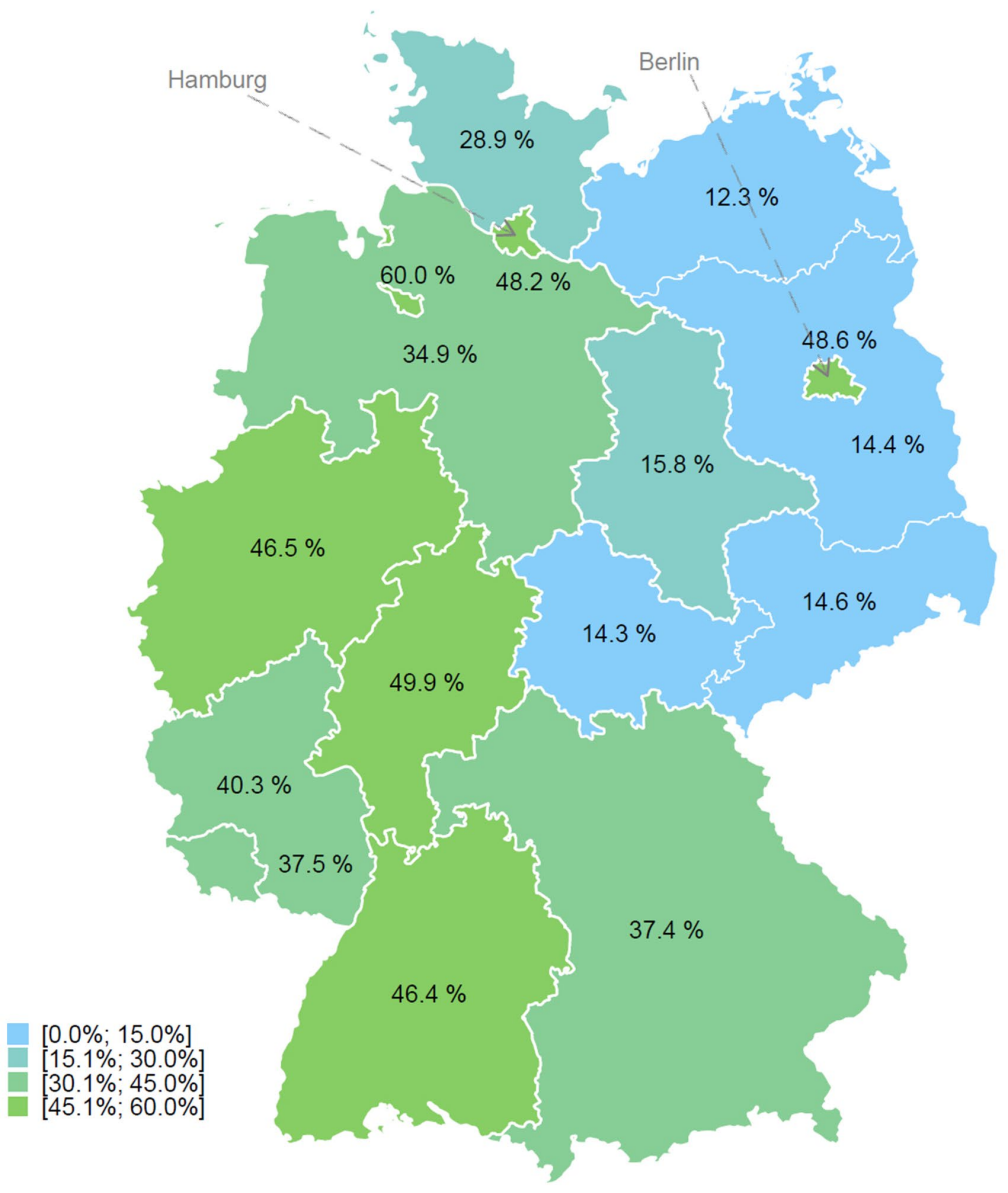
Proportion of the population with a migrant background in the 0 to 17 age group by federal state in Germany in 2019; Own representation based on data from the Federal Statistical Office (3).

The intervention to increase the use of medical rehabilitation by children and adolescents with a migrant background is based on previous campaigns of the Ethno-Medical Center e. V. (EMC) (18, 19). The assumption that migrants in health care provision should not only be seen as the target of advice and support, but as central actors in their own social and health-related integration with genuine resources and competencies is central to the project. The project is funded by German Pension Insurance Berlin-Brandenburg (GPI BB) and German Pension Insurance North (GPI North). The intervention consists of four modules: 1. mediator training (Module 1), 2. information events (Module 2), 3. individual support for rehabilitation requests (Module 3), and 4. transcultural training for health professionals (Module 4). The individual modules are described in detail below. The implementation takes place in the two cities of Berlin and Hamburg. These cities were chosen because they have a particularly high proportion of people with a migrant background (Figure 1), and a high population density due to their urban structure, which facilitates the feasibility of the individual modules within the framework of the limited financial and human resources in the trial (3).

The implementation is accompanied by a process evaluation (20). The effect of the intervention (i.e., increase in utilization of MRCA by children and adolescents with a migrant background) will be tested with a repeated cross-sectional survey (trend study) with four measurement points. The information campaign will be implemented by the Ethno-Medical Center. The process evaluation and the trend study are both implemented by the Institute of Social Medicine and Epidemiology of the University of Lübeck (UoL). The implementation of the information campaign, the implementation of the trend study and the process evaluation are accompanied by an advisory board, which consists of responsible persons from the GPI BB and GPI North sponsors as well as migration experts and specialists from the fields of pediatric and adolescent medicine and medical rehabilitation. The advisory board meets every six months and uses its expertise to address problems that arise during the implementation. The project started in April 2019, and once the campaign module design and materials development were completed, the campaign modules began implementation in September 2020. Implementation of the campaign modules and process evaluation will continue to the end of 2022. The final survey of the trend study will take place at the end of 2023.

### 2.2. The intervention and underlying assumption

Health promotion interventions tailored to the needs of subgroups or communities within an overall population may be better suited to addressing health inequalities within a population (21, 22). Accordingly, the WHO also recommends the greater involvement of migrants and migrant communities in health promotion interventions in order to strengthen their personal health literacy and reduce inequalities (17). The intervention described here follows these principles. The intervention builds on the EMC’s concept “MiMi – With Migrants, For Migrants” (18, 19). The concept follows a setting approach, which sees the consideration and inclusion of the social environment as a central aspect of successful measures to improve health behavior (23). In the context of MiMi-Reha-Kids, this means taking appropriate account of the heterogeneous social environment of migrants in Germany, and actively involving migrants in the process of health promotion. In order to ensure this, MiMi-Reha-Kids follows an empowerment strategy by training people from migrant communities to become transcultural mediators, who then act as multipliers in their communities, conducting information events in their native language and supporting families in requesting medical rehabilitation for children and adolescents.

Migrants are thus actively involved in local health promotion, and knowledge is anchored in migrant communities. Information can be passed on according to the specific needs of people with MB, since the members of the communities can be seen as experts for their own social environment, and therefore know best who can be addressed where, when and how in a target group-oriented manner, and informed about the topic of medical rehabilitation for children and adolescents. Together with external instructors, the project managers conduct the initial training sessions and develop multilingual materials. These materials will be used by the mediators to conduct the information events and support rehabilitation requests. Further training on transcultural competence is also offered for health care professionals in order to raise awareness about the specific needs of families with migrant backgrounds. During the initial phase of intervention development, qualitative interviews will be conducted with parents with a history of migration and a chronically ill child, as well as chronically ill adolescents themselves, in order to identify additional barriers and challenges in accessing medical rehabilitation and to tailor the modules of the information campaign accordingly. Additional file 1 describes the four modules according to the Template for Intervention Description and Replication (TIDieR) (24).

#### 2.2.1. Mediator training (Module 1)

The intervention starts with the acquisition of future transcultural mediators from different migrant communities (e.g., Turkish community) at the project sites in Berlin and Hamburg. Contact and acquisition is through the EMC network, and considers the social environment of the migrants. Potential mediators are recruited, for example, in religious, social, and migrant self-organizations, cultural centers or in educational institutions using flyers, posters, or by word of mouth. The recruitment targets individuals with advanced German skills and extended networks in the respective migrant community. The mediators are provided with knowledge about the general and health situation of migrant families in Germany, and about the German health care system, in extensive training courses (Module 1), with a special focus on medical rehabilitation, its goals, contents and possible uses. The mediators are also taught didactic and methodological skills in the training sessions, which enable them to pass on their acquired knowledge to other members of the migrant communities as multipliers in independently organized information events. The entire training comprises 48 teaching units of 45 min each, which are distributed over nine training days. The sessions are led by instructors with subject-specific experience (e.g., pediatricians on the topic of chronic diseases in children). Participants receive a training folder with prepared information on the training content. On the last day of training, the trained mediators give a presentation in small groups on one of the topics covered in the training (e.g., requesting on medical rehabilitation for children and adolescents) as a final exam. A total of at least 120 mediators (60 in Berlin and 60 in Hamburg) will thus be trained in three years from 2020 to 2022.

#### 2.2.2. Information events in the native language (Module 2)

After completing the training, the mediators inform migrants about medical rehabilitation for children and adolescents in independently conducted information events in their native language (Module 2). In addition to their training folder, the trained mediators receive other support materials, such as a multilingual guide to medical rehabilitation for children and adolescents (see Additional file 2) that can be handed out to interested parents and adolescents, and a PowerPoint presentation that they can use for the information session. Both the guide and the presentation present the contents of the training in a condensed and easy-to-understand form. In addition to a German-language version, these materials are available in Arabic, Bulgarian, English, Farsi, Italian, Kurdish, Polish, Russian, Spanish, Serbian/Croatian/Bosnian and Turkish, so that information events can be held in the native language of parents and interested parties. The information events should have a duration of about 120 min, and be carried out with about 10 participants. The information events can take place at different locations in the migrants’ social environment, for example in migrant cultural associations, but also in doctors’ offices, schools or EMC venues. The mediators are responsible for organizing the sessions themselves, but receive support from the EMC if necessary. The mediators receive a financial compensation for each information session held. Overall, it is planned that the mediators will hold a total of 240 information events by the end of 2022, thus informing around 2,400 people in Berlin and Hamburg about the possibilities of medical rehabilitation.

#### 2.2.3. Individual support for rehabilitation requests (Module 3)

In addition to information events, the trained mediators offer individual support for rehabilitation requests (Module 3). Families with chronically ill children can receive advice in their native language on the process for requesting medical rehabilitation or filling out the application forms with the support of the mediators. The mediators can use a written guide on how to apply for medical rehabilitation for children and adolescents, which was designed within the project. The counseling can take place in a public location, at the home of interested persons, or in venues of the EMC. The counseling service is also particularly important because application forms for medical rehabilitation in Germany are currently only available in German. The mediators receive financial compensation for each consultation on a rehabilitation request. A total of 48 individual consultations are to be carried out by the mediators.

#### 2.2.4. Transcultural training for professionals (Module 4)

Transcultural competence, i.e., culture-related knowledge, sensitivity toward different life worlds and the ability to critically reflect on one’s own sociocultural location (25), is a key-competence in achieving better outcomes in health-related interaction and communication among individuals with different sociocultural backgrounds (26). The project therefore also aims to promote the transcultural competence of health professionals. A total of at least four all-day training courses on transcultural competence and communication (Module 4) will be held in 2021 and 2022 in Berlin and Hamburg for health professionals from the field of rehabilitative care who are either directly involved in the implementation of medical rehabilitation (e.g., therapists and nurses from rehabilitation centers), or have a steering function in the process of medical rehabilitation for children and adolescents (e.g., pediatricians). The training sessions are offered by external certified coaches for transcultural communication. By raising awareness on barriers specific to the project target group (e.g., language-and culture-based barriers), the trainings strengthen the integrative competence of health professionals when counseling and treating persons from migrant backgrounds. There is no fee to attend.

### 2.3. Evaluation design

**Figure 2:**
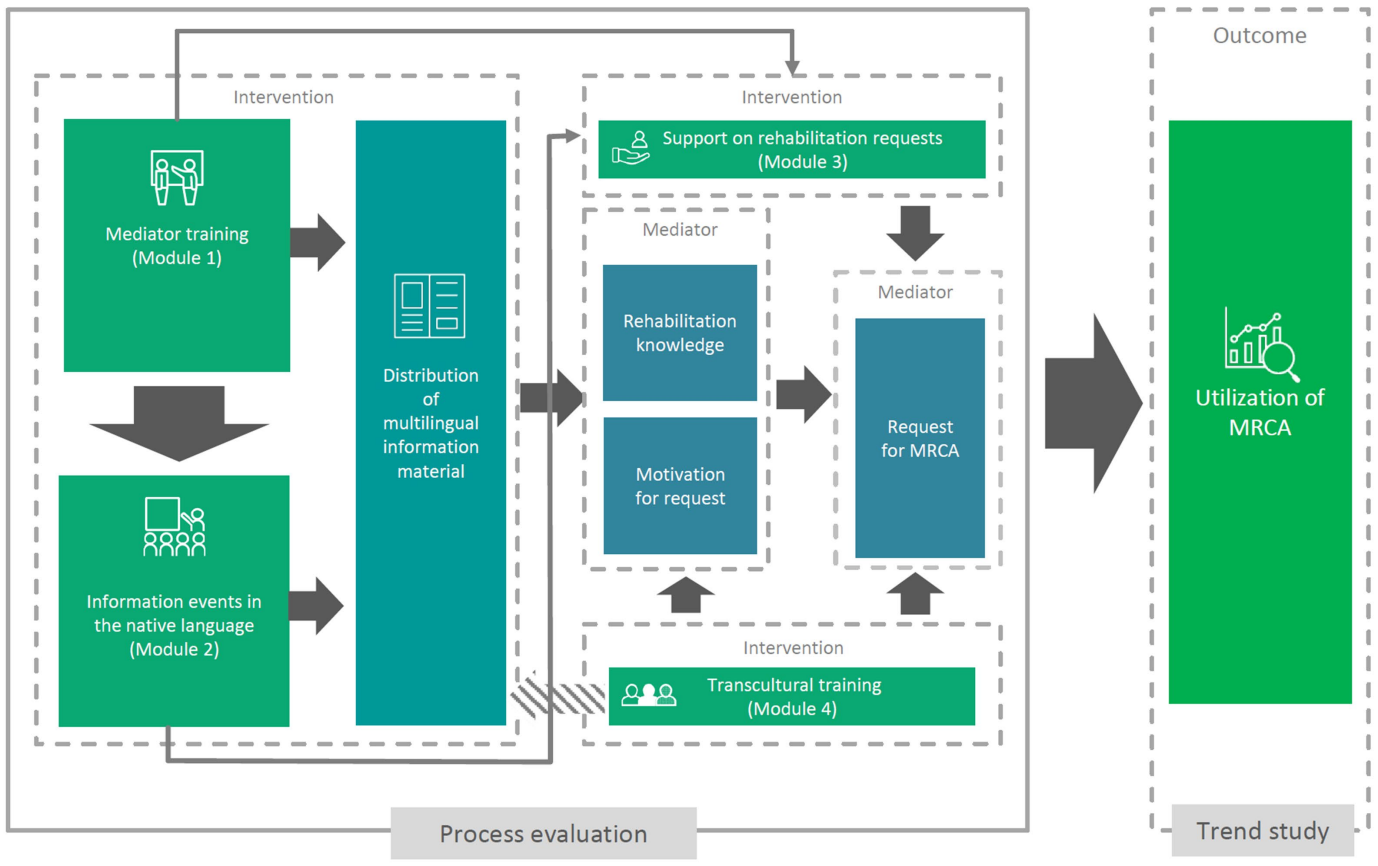
Potential mechanism of impact for the Multilingual Information Campaign on Rehabilitation for Children and Adolescents with Migrant Background.

In order to evaluate the intervention, we combine a trend study consisting of a repeated retrospective cross-sectional survey (27) at four measurement time points (2020, 2021, 2022, 2023), with an accompanying process evaluation of the information campaign. The process evaluation is based on the recommendations of the British Medical Research Council for complex interventions (20). The evaluation of complex interventions faces the challenge that when outcomes are being assessed it is not always immediately determinable which elements have contributed to success or non-success in the sense of describable effects on the outcome, due to the multiplicity of different elements and their possible interactions (28). A description of the assumed mechanism of action of the intervention together with an evaluation of the implementation of the individual intervention elements (e.g., reach, dose delivered, dose received), can help to better understand which factors of the complex intervention may have affected the outcome (28). Knowledge about implementing the different modules is not only important to explain observed outcomes, however, but also with regard to the further development or adaptation of the intervention in other settings. The potential mechanism of impact for the MiMi-Reha-Kids intervention and the interaction of the different modules is shown in Figure 2.

As part of the process evaluation, questionnaires will be used to continually collect data on the implementation of the individual modules of the intervention during the active implementation of the information campaign from October 2020 to December 2022. The data collected describes, for the different modules mentioned above, and among other things, the scope of the sessions (duration, frequency), the number of participants, the socio-demographics of the participants, implemented content, the subjective assessment of the information gain, and challenges of implementation. Data will be collected from both participants and instructors (Table 1). The effectiveness of the intervention will be tested by a trend study with repeated retrospective written cross-sectional surveys in the project sites of Berlin and Hamburg of families in which a child lives who received medical rehabilitation from German Pension Insurance in the previous year, in order to describe the change in the proportion of rehabilitants with a migrant background. The first cross-sectional survey was conducted in the fourth quarter of 2020, and is used to describe the proportion of children and adolescents with a migrant background in 2019 and thus before the implementation of the information campaign. The last retrospective cross-sectional survey will take place in the fourth quarter of 2023. The questionnaire data will be linked to the administrative data of the applicants.

**Table 1:** Description of the process evaluation tools and elements.

Module	Process evaluation tool	Process evaluation element	Information/Data
Mediator training (Module 1)			
	Recruitment monitoring	Recruitment/reach	Number of mediators per training
	Documentation sheet for instructors	Recruitment/reach	Number of mediators per training unit
		Fidelity	Implementation of the module in comparison to the conceptual design (e.g., duration, contents); Feedback from instructors on implementation problems with closed and open-ended questions
		Dose delivered	Duration of the training course
			Materials used
			Subjective evaluation of the training course implementation from the instructor’s point of view
	Questionnaire 1 for mediators (at each individual appointment)	Dose received	Subjective evaluation of the implementation of the training date
			Recommendations of the mediators to improve training
	Questionnaire 2 for mediators (after completion)	Recruitment/reach	Sociodemographic data of the mediators
		Dose delivered	Data on attendance at the individual training dates
		Dose received	Subjective evaluation of the implementation of the entire training
			Recommendations of the mediators to improve training
Information events in the native language (Module 2)			
	Documentation sheet for mediators	Recruitment/reach	Number of participants
		Fidelity	Implementation of the module in comparison to the conceptual design (e.g., duration, contents); Feedback from mediators on implementation problems with closed and open-ended questions
		Dose delivered	Location of the information session
			Duration of the information session
			Information session language
			Sociodemographic data on the mediator performing the mediation
			Materials used
			Information on topics covered
			Subjective evaluation of implementation of the information session from the mediator’s point of view
	Questionnaire for participants in twelve languages	Recruitment/reach	Sociodemographic data of the participants
		Dose received	Subjective evaluation of implementation of the information session
Individual support for rehabilitation requests (Module 3)			
	Monitoring of the implementation	Dose delivered	Number and duration of consultations
		Recruitment/reach	Number of participants, language of consultations
	Documentation sheet for mediators	Fidelity	Implementation of the module in comparison to the conceptual design (e.g., duration, contents); Feedback on implementation problems with closed and open-ended questions
Transcultural training for professionals (Module 4)			
	Recruitment monitoring	Recruitment/reach	Number of participants
	Documentation sheet for instructors	Fidelity	Implementation of the module in comparison to the conceptual design (e.g., duration, contents); Feedback from instructors on implementation problems with closed and open-ended questions
		Dose delivered	Training duration
			Subjective evaluation of implementation of the training from the instructor’s point of view
	Questionnaire for participants of the training	Recruitment/reach	Sociodemographic data of the participants
		Dose received	Subjective evaluation of implementation of the training from the point of view of the participants

### 2.4. Inclusion criteria

#### 2.4.1. Trend study

The sample for the repeated cross-sectional survey of the trend study consists of all insured persons with the Federal German Pension Insurance, German Pension Insurance Berlin-Brandenburg, and German Pension Insurance North residing in Berlin or Hamburg whose child received medical rehabilitation through the German Pension Insurance in 2019, 2020, 2021, or 2022 and were aged from 0 to 17 years at the time of utilization. These three providers collectively account for the majority of the insured population in the two model regions, making it possible to include nearly all eligible families in the sample.

#### 2.4.2. Processes evaluation

As part of the process evaluation, all participants and instructors of the four campaign modules are asked to complete the questionnaires at the end of the modules or sessions.

### 2.5. Outcomes and other measures

#### 2.5.1. Trend study

The primary outcome of the trend study on the utilization of rehabilitation is the proportion of children and adolescents with a migrant background among those undergoing rehabilitation who reside in Berlin or Hamburg. The migrant background is assessed in this study in accordance with the minimum indicator set for recording migration status (29).

We determine an individual as having a migrant background if they immigrated from another country and have at least one parent who was not born in Germany, or have two parents who immigrated and/or do not have German nationality.

This definition allows a differentiation between children with unilateral and bilateral migrant backgrounds (30). The information on the country of birth and nationality of the parents and the child required to map the migrant background is assessed through a questionnaire, and combined with data from the insured person’s account. The primary language, residence status, and duration of residence of the child and the child’s parents are also assessed.

In order to provide a description of the samples and to describe differences between children and adolescents with and without a migrant background in the samples, the health-related quality of life of the children and adolescents [KIDSCREEN-27 (31)], sociodemographic and socioeconomic information on the children and the parental home (32), perceived social support (33) and satisfaction with the rehabilitation service received will be collected through questionnaires as part of the trend study. The questionnaire data is supplemented by administrative data on medical rehabilitation (e.g., duration, and discharge diagnosis according to ICD-10) from parents’ insurance accounts. The data collected in the trend study is shown in Table 2.

**Table 2:** Data collected as part of the trend study.

Data	Source^2^	Sample 2020	Sample 2021	Sample 2022	Sample 2023
Migration history (34)		x	x	x	x
Nationality^1^	Questionnaire for parents/administrative data	x	x	x	x
Country of birth^1^	Questionnaire for parents	x	x	x	x
Primary language^1^	Questionnaire for parents	x	x	x	x
Duration of stay^1^	Questionnaire for parents	x	x	x	x
Residence status^1^	Questionnaire for parents	x	x	x	x
Socioeconomic status (31)	Questionnaire for parents	x	x	x	x
Social support (32)	Questionnaire for parents	x	x	x	x
Health-related quality of life (KIDSCREEN-27) (30)		x	x	x	x
Physical wellbeing	Questionnaire for parents/child	x	x	x	x
Psychological wellbeing	Questionnaire for parents/child	x	x	x	x
Parent relations and autonomy	Questionnaire for parents/child	x	x	x	x
Social support and peers	Questionnaire for parents/child	x	x	x	x
School	Questionnaire for parents/child	x	x	x	x
Satisfaction with rehabilitation	Questionnaire for parents/child	x	x	x	x
Start of rehabilitation	Administrative data	x	x	x	x
End of rehabilitation	Administrative data	x	x	x	x
Approval diagnosis (ICD-10)	Administrative data	x	x	x	x
Discharge diagnosis (ICD-10)	Administrative data	x	x	x	x

^1^Data is collected for the child and both birth parents. ^2^The parent questionnaire is sent to the parent who requested the child’s medical rehabilitation, and includes a section for information about the other parent; Administrative data is extracted from the insurance account of the parent who applied for medical rehabilitation.

#### 2.5.2. Process evaluation

The process evaluation takes into account recommendations from Saunders et al. for developing and describing complex interventions (34). Accordingly, the components recruitment/reach, fidelity, dose delivered, and dose received will be assessed using different questionnaires and documentation forms as part of the process evaluation for the modules of the intervention (Table 1).

### 2.6. Sample size

#### 2.6.1. Trend study

A sample of 432 persons per year must be recruited in order to be able to demonstrate a change in the proportion of children and adolescents with a migrant background among the rehabilitants from 10 to 11%, 13, and 16% in the trend study [Cochran-Armitage test for trend (35)]. With a participation rate of 40% and 1,200 children and adolescents residing in Berlin or Hamburg receiving rehabilitation, power of 84% is needed to detect a significant trend. If the participation rate for persons without a migrant background is higher than for persons with a migrant background, the total sample size increases. Despite the observed reduced proportions of children and adolescents with a migrant background, the strength of the Cochran-Armitage test for trend is almost unchanged for the then-expected reduced trend.

#### 2.6.2. Process evaluation

No sample size calculation was made for the process evaluation. We intend to recruit 120 participants for mediator training (Module 1) and 2,400 participants in the information events (Module 2). We expect 48 participants for individual counseling on requests for medical rehabilitation (Module 3), and 60 participants from the health care sector in transcultural training (Module 4).

### 2.7. Recruitment

#### 2.7.1. Trend study

In the fourth quarter of 2020 to 2023, the German Pension Insurance identifies, based on administrative data, all insured persons residing in Berlin or Hamburg who have a child who received medical rehabilitation from the German Pension Insurance in the previous year (i.e., 2019, 2020, 2021, and 2022) and who was 17 years old or younger at the time of rehabilitation. These insured persons form the sample of the trend study.

#### 2.7.2. Process evaluation

People who participate in a session within the framework of the four modules during the information campaign are asked to complete questionnaires on site. Instructors document the sessions on documentation sheets.

### 2.8. Data collection

#### 2.8.1. Trend study

Trend study data will be generated through questionnaires and from administrative data from insured accounts. Insured persons identified by the German pension insurance will receive a cover letter, study information, a parent questionnaire, a child questionnaire (for the child who received medical rehabilitation), and a consent form for the use of administrative data at the beginning of the 4th quarter of 2020, 2021, 2022, and 2023. The questionnaires are pseudonymized. The questionnaires are returned to the University of Lübeck by the insured persons and electronically recorded there. The declaration of consent is sent by the insured persons to the German Pension Insurance, who send one reminder after four weeks. If consent is given, German pension insurance sends a pseudonymized data set with data from the insured persons’ accounts to the University of Lübeck, where this administrative data is linked to the questionnaire data using the pseudonym.

#### 2.8.2. Process evaluation

Participants receive an anonymous questionnaire at the end of a session, which is completed on site and submitted to the instructors. The documentation and questionnaires for Modules 1, 3 and 4 are available in German. The questionnaires for participants in information events in their native language (Module 2) are available in twelve languages (see above), and are distributed according to the language of the participants. The instructors send the questionnaires, together with the completed documentation forms, by mail to the University of Lübeck, where all data is recorded electronically.

### 2.9. Data management

A comprehensive data protection concept was developed in coordination with German pension insurance, which ensures data processing, the rights of the persons involved, as well as the technical and organizational measures for the secure and confidential collection, processing and storage of data. Researchers at the University of Lübeck only receive pseudonymized data from the insured persons and their children as part of the trend study. The generation of data within the process evaluation is anonymized. All questionnaire data is entered, checked and transferred to statistical software programs for further analysis. Data entry and data verification are performed by trained research assistants. Questionnaire data from the trend study is linked to administrative data from the insurance account using a pseudonym. Access to the data is limited to the authors and research assistants on the research team.

### 2.10. Statistical methods

Changes in the proportion of individuals with a migrant background in the samples will be analyzed using a Cochrane-Armitage test for trend (36). We will use unpaired t-tests for metric data, a Mann–Whitney U-test for ordinal data, and chi-square test for nominal data to describe differences between rehabilitants with and without migrant backgrounds in the samples. We will not perform interim analyses and did not specify a stopping rule. The results of statistical tests will be considered significant if the two-sided value of p of the test is less than 0.05. Process evaluation data is descriptively presented. A current version of Stata (StataCorp, College Station, Texas, USA) will be used to perform the analyses.

## 3. Discussion

The aim of our evaluation is to describe the implementation of a complex multimodal information campaign to improve the utilization of medical rehabilitation by children and adolescents with a migrant background, and to test the effectiveness of this intervention. The intervention aims to improve health-related support and information provision for a particularly vulnerable population group, and thus to make a lasting contribution to reducing social inequality in health care in Germany. The intervention presented here is a participatory measure that actively involves migrants in the process of providing information, in line with recent WHO recommendations (17), and aims to sustainably anchor the knowledge and skills imparted in migrant communities. The process evaluation and trend study will generate data that will allow a detailed assessment of the implementation and success of the described information campaign. In this way, the project will enable the intervention to be adapted and further developed for similar objectives, for example with regard to other healthcare services or other regions. Up-to-date information on the information campaign and the study can be found on the website www.mimi-reha-kids.de. The results of our study will all be published as articles in peer-reviewed journals and at conferences. The authors of this protocol will write the final study publications. The use of professional authors is not anticipated.

## 4. Conclusion

The study described in this protocol will provide comprehensive data on which persons can be reached within the different modules of the multimodal information campaign and how the information campaign and its different modules are perceived by the participants. The results of the trend study will also provide information on whether the utilization of medical rehabilitation by children and adolescents with a migrant background could be increased over the intervention period at the intervention sites. Thus, possible changes in utilization can be related to the specific implementation of the modules of the information campaign. These data can help organizations and institutions to adapt or further develop the multimodal information campaign or specific modules for other regions in Germany or other countries.

## Trial status

Process evaluation and cross-sectional surveys of the trend study have begun and will continue.

## Ethics statement

The studies involving human participants were reviewed and approved by Ethics Committee of the University of Lübeck. Written informed consent to participate in this study was provided by the participants’ legal guardian/next of kin.

## Author contributions

MB and HB developed the study design and conducted the evaluation. RS and FB conceived the information campaign with contributions from MB and HB and involved in the practical implementation of the intervention. HB prepared the first draft of the manuscript with contributions from MB, FB, and RS. All authors read and approved the manuscript before submission and fulfil the authorship criteria of the International Committee of Medical Journal Editors.

## Funding

The study was funded by German Pension Insurance Berlin-Brandenburg, Bertha-von-Suttner-Str. 1, 15236 Frankfurt (Oder), Germany and German Pension Insurance North, Ziegelstraße 150, 23556 Lübeck, Germany. Funding includes personnel, material and travel costs. German Pension Insurance Berlin-Brandenburg, federal German Pension Insurance, and German Pension Insurance North also contributed to the study by sending study materials and questionnaires and by providing administrative data. We acknowledge financial support by Land Schleswig-Holstein within the funding program Open Access Publikationsfonds. The funding bodies have no influence over the study design, data collection, data analysis, data interpretation, or writing of the manuscript. Anonymous data processing and data analysis are guaranteed.

## Conflict of interest

The authors declare that the research was conducted in the absence of any commercial or financial relationships that could be construed as a potential conflict of interest.

## Publisher’s note

All claims expressed in this article are solely those of the authors and do not necessarily represent those of their affiliated organizations, or those of the publisher, the editors and the reviewers. Any product that may be evaluated in this article, or claim that may be made by its manufacturer, is not guaranteed or endorsed by the publisher.

## Abbreviations

EU: European Union
EMC: Ethno-medical Center
GPI: German Pension Insurance
ICD: International Classification of Diseases
IOM: International Organization for Migration
MB: Migrant background
MRCA: Medical Rehabilitation for Children and Adolescents
OECD: Organization for Economic Co-operation and Development
WHO: World Health Organization
SGB: Sozialgesetzbuch (Social Security Code)
TIDieR: Template for Intervention Description and Replication
