# Supplementary material for: Increasing the use of medical rehabilitation by children and adolescents with migrant background through a multimodal information campaign: protocol of a trend study and accompanying process evaluation (MiMi-Reha-Kids, DRKS00019090)
Source: Front Public Health. 2023 Jul 14;11:1089685. doi: 10.3389/fpubh.2023.1089685 (PMC10379645; doi:10.3389/fpubh.2023.1089685)
Supplement: Supplementary file 1 [file Data_Sheet_1.PDF]

**Additional file 1: Description of the four components according to the TIDieR checklist**

| Brief name | Mediator training<br>(Module 1) | Information events in the native language<br>(Module 2) | Individual support for rehabilitation requests<br>(Module 3) | Transcultural training<br>(Module 4) |
|---|---|---|---|---|
| Why | According to experts, children and adolescents with a migrant background make less use of medical rehabilitation than their peers without a migrant background, despite a comparable need based on epidemiological figures. The WHO recommends the inclusion of migrants and migrant communities in measures to reduce inequalities in health and health care, in order to strengthen the personal health literacy of migrants and to anchor knowledge in migrant communities. Against this background, the MiMi-Reha-Kids project implements a multimodal and multiplier health campaign, the first module of which is the training of so-called transcultural mediators from the migrant communities, who are to pass on the acquired knowledge as multipliers. | The information events on medical rehabilitation represent the second module and are organized by the trained mediators. Compared to the provision of information by institutions such as the German Pension Insurance, they offer low-threshold access in a migrant's language of origin in a familiar social space. | Due to the complexity of the medical rehabilitation request process, the information provided in the information events is not sufficient in some cases to enable participants to apply for rehabilitation. Furthermore, individuals may have very specific or personal questions that cannot or should not be answered in the group. In this case, people have the option of seeking individual support for rehabilitation requests from the mediators. | Transcultural competence is a tool with which to achieve better outcomes in health-related interactions and communication between persons with different sociocultural backgrounds. Transcultural training for health professionals from rehabilitative care settings will therefore be offered as a complementary component of the project. |
| What (materials) | Training folder with the collected contents of the training on medical rehabilitation for children and adolescents. | Training folder; guide (brochure) on medical rehabilitation in child and parent versions for distribution to participants (in 12 languages); PowerPoint | Training folder; guide to applying for medical rehabilitation for children and adolescents in the form of a brochure; guide (brochure) to medical | Written handout on transcultural communication for participants. |

| | | | | |
|---|---|---|---|---|
| | | presentation with key information on medical rehabilitation for children and adolescents (e.g. indications, legal requirements, request, rehabilitation process) in 12 languages. | rehabilitation in child and parent versions for distribution to those seeking advice (in 12 languages). | |
| What (procedures) | The Ethno-medical Center e. V. uses its contacts with various migrant organizations to recruit well-connected multilingual migrants (German and at least one other language) with an interest in providing health information for the training sessions. The participants go through several days of training in which they receive information about the life situation of migrants in Germany, the German health care system and medical rehabilitation (indication, legal requirements, request, etc.). Migrants also learn in units on didactics and methodology to independently implement information sessions. The training is concluded with an examination in the form of a presentation on the topic of medical rehabilitation for children and adolescents. | The trained mediators promote information sessions on medical rehabilitation for children and adolescents through their personal contacts in the migrant communities and with the support of the Ethno-medical Center e. V. The information events are offered in the native language of the attending migrant group. The mediators are provided with a PowerPoint presentation containing the most important information on medical rehabilitation for children and adolescents in 12 languages. Depending on the preferences of participants and the mediators, the event takes place at different locations such as cultural associations, migrant organizations or religious institutions (mosques, churches). At the end of the information event, the mediators hand out written guides (brochure) to interested parties and refer to the possibility of individual support for rehabilitation requests. | People seeking advice meet with the mediator at an agreed location for individual support for rehabilitation requests. In addition to their training materials, mediators are provided with a guide for submitting a request for medical rehabilitation through the German pension insurance. | The Ethno-medical Center e. V. advertises transcultural training via various channels (newsletter, flyer, direct approach), and books a certified coach for transcultural communication for the implementation. In a full-day seminar, participants are trained in dealing with diversity in the healthcare sector and also receive a step-by-step handout on the topic. |
| Who provided | Ethno-medical Center e. V. and topic-specific external instructors | Trained mediators | Trained mediators | Certified trainers for transcultural communication. |

| | | | | |
|---|---|---|---|---|
| | (e.g. pediatricians, rehabilitation counselors). | | | |
| How | In groups with 15 to 25 participants; usually face-to-face. | In groups with 5 to 15 participants; usually in presence. | Individual; in presence. | In groups with 15 to 25 participants; in presence. |
| Where | Rooms of the Ethno-Medical Center e. V. in Berlin and Hamburg. | Different locations depending on the choice of mediators (e.g. cultural associations, charitable institutions) in Berlin and Hamburg. | Different locations depending on the choice of mediators and people seeking advice (e.g. cultural associations, private households) in Berlin and Hamburg. | Rooms of the Ethno-Medical Center e. V. in Berlin and Hamburg. |
| When and how much | In 2020, 2021 and 2022 respectively; 48 teaching units of 45 minutes each over 9 training days; training of a total of 120 mediators. | 120 minutes; a total of about 240 information sessions with 2400 participants. | 48 individual support sessions on rehabilitation requests lasting approximately 1 hour per appointment. | At least 4 training sessions of full-day duration; at least 60 participants in total. |
| Tailoring | Not planned. | The information events take into account the specific living environment of the participants with regard to their information needs (e.g. language, illness of the child). | The individual support for rehabilitation requests takes into account the request-specific needs of those seeking advice (e.g. objection, initial request). | Not planned. |
| How well | Questionnaires to trainers on the organizational and content implementation of each unit; questionnaires to participants on implementation and satisfaction after each unit and at the end of training. | Written documentation of formal and substantive implementation of each event by mediators; questionnaires to participants on implementation and satisfaction (in language of origin). | Written documentation of the implementation of each individual support for rehabilitation requests by mediators. | Questionnaires to instructors on the organizational and content-related implementation of the training; questionnaires to the participants on the implementation and satisfaction at the end of the training. |
