# Supplementary material for: Increasing the use of medical rehabilitation by children and adolescents with migrant background through a multimodal information campaign: protocol of a trend study and accompanying process evaluation (MiMi-Reha-Kids, DRKS00019090)
Source: Front Public Health. 2023 Jul 14;11:1089685. doi: 10.3389/fpubh.2023.1089685 (PMC10379645; doi:10.3389/fpubh.2023.1089685)

Englisch

Kompetenz-Zentrum
Rehabilitation der DRV

MiMi Mit Migranten
für Migranten

Version for parents/
Version für Eltern

Medical rehabilitation through German Pension Insurance:

Support for children and adolescents with chronic illness

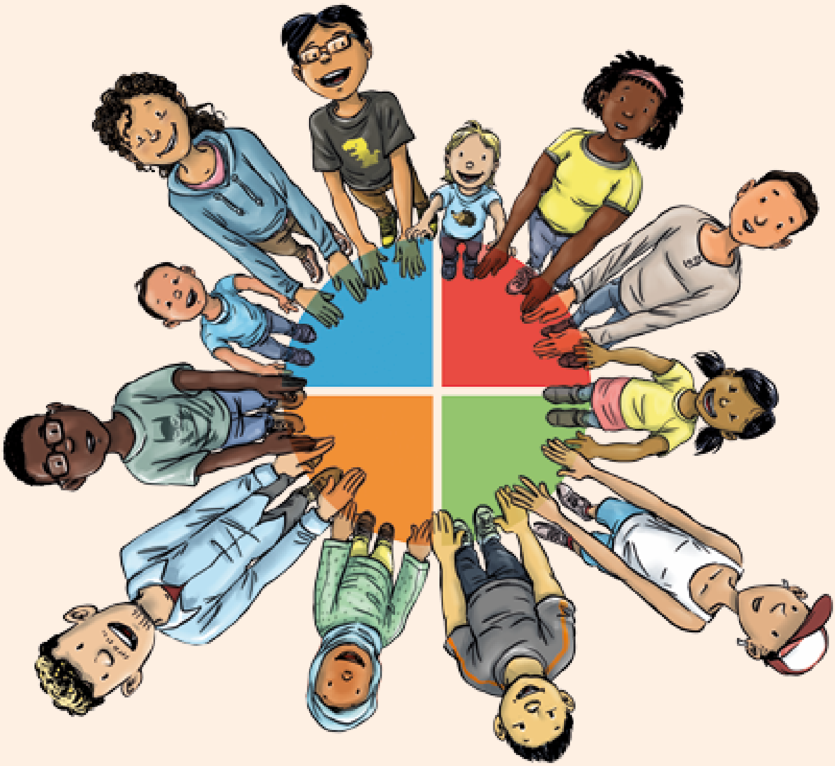

A guide for migrants. Available in 10 languages.

Deutsche Rentenversicherung Nord

Deutsche Rentenversicherung Berlin-Brandenburg

Universität zu Lübeck

Ethno-Medizinisches Zentrum e.V.

Impressum

Die medizinische Rehabilitation der Deutschen Rentenversicherung:
Hilfe für chronisch kranke Kinder und Jugendliche
Ein Wegweiser für Migrant*innen

Herausgeber – Konzeption, Inhalt, Erstellung:
Ethno-Medizinisches Zentrum e.V.
Königstraße 6 · 30175 Hannover

Mitarbeit:
Universität zu Lübeck
Institut für Sozialmedizin und Epidemiologie
Ratzeburger Allee 160 · 23538 Lübeck

Förderung:
Deutsche Rentenversicherung Nord und Deutsche Rentenversicherung Berlin-Brandenburg

Wissenschaftliche Projektleitung:
Ramazan Salman, Ethno-Medizinisches Zentrum e.V.
Prof. Dr. Matthias Bethge, Univ. zu Lübeck, Inst. f. Sozialmedizin u. Epidemiologie

Redaktion: Ramazan Salman, Prof. Dr. Matthias Bethge, Lisa Ohmes, Hannes Banaschak, Jasmin Kreth, David Peter Fauser, Anette Metzger, Deborah Amoah, Olga Kedenburg, Elena Kromm-Kostjuk, Anne Rosenberg, Soner Tuna, Ali Türk, Michael Weig, Eva Renckly-Senel, Ahmet Kimil

Übersetzung: Dolmetscherdienst Ethno-Medizinisches Zentrum e.V.

Bildquellen: Titelbild und Illustrationen © Raimund Frey, Porträt Christian Wolff © DRV Berlin-Brandenburg, Porträt Dr. Ingrid Künzler © Dietmar Theis, Abb. 1, 2, 3: eigene Darstellung

Satz und Layout: eindruck.net

Alle Rechte vorbehalten. Das Werk ist urheberrechtlich geschützt. Jede Verwendung in anderen als den gesetzlich zugelassenen Fällen bedarf deshalb der vorherigen schriftlichen Genehmigung durch den Herausgeber.

1. Auflage

Dieser Wegweiser ist in folgenden Sprachen erhältlich:
Arabisch, Bulgarisch, Deutsch, Englisch, Farsi/Persisch, Kurdisch (Kurmandschi), Polnisch, Russisch, Serbisch/Kroatisch/Bosnisch und Türkisch.

Stand: Juni 2020

Medical rehabilitation through
German Pension Insurance:

Support for children and adolescents with chronic illness

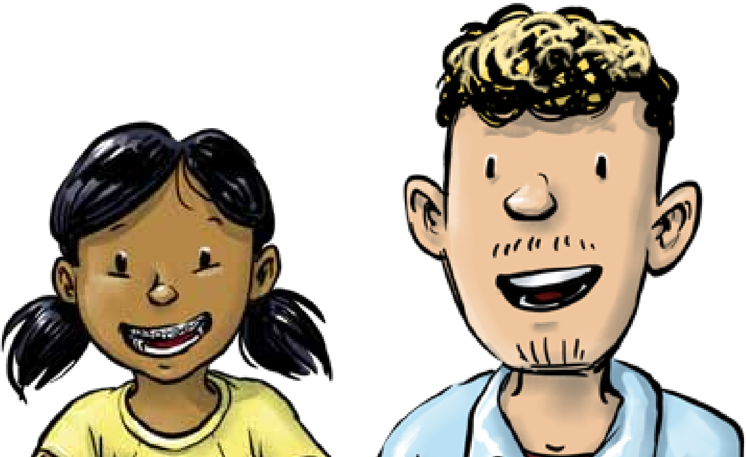

Welcome message

Dear parents,

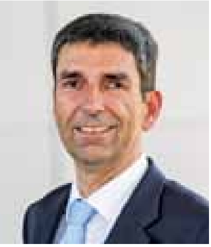

Children and adolescents deserve the best possible protection and the best health care. With its medical rehabilitation services, German Pension Insurance (Deutsche Rentenversicherung) contributes to these goals for children and adolescents with long-term, often chronic illness.

According to the professionals, children with migration experiences do not speak German at home rarely use medical rehabilitation services. It is assumed that this is due to a lack of information and awareness of the opportunity to access these benefits, as we as parents' language difficulties. On the other hand, medical rehabilitation as an institutionalised part of the health system needs to become more transculturally accessible in order to attract linguistically, religiously and culturally diverse groups of patients.

The Mimi-Reha-Kids project tackles this issue from both ends. It conducts transcultural professional development courses for specialist personnel and provides multilingual information for affected families at the same time. This guide offers you comprehensive information on the topic of medical rehabilitation for children and adolescents. There are two versions of this guide: one for parents of children with long-term illness and one for young people who want to find out information for themselves. It is available in ten languages, offering many affected families a good foundation for obtaining an overview of the medical rehabilitation services on offer.

I would like to express my gratitude to the staff at the Ethno-Medical Centre Inc. (Ethno-Medizinisches Zentrum e.V.) and at Lübeck University (Universität zu Lübeck) for developing this guide. I would also like to express my appreciation for the work of those who are taking care of the translations. Offering multilingual information provides valuable support to all those who are affected on their path to taking up medical rehabilitation services for children and adolescents.

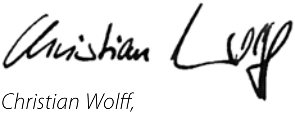

Christian Wolff,
Deputy Executive Officer, German Pension Insurance (DRV) Berlin-Brandenburg

Welcome message

Dear parents,

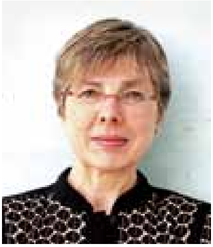

chronic illness imposes a significant burden on everyday life. If children or adolescents are affected by chronic illness, they often have fewer opportunities in terms of their participation in the education system. Because of their illness, they may also face difficulties in everyday life and in their social relations. In this context, medical rehabilitation services represent a great opportunity for children and adolescents with chronic illness.

The treatment that young patients receive at rehabilitation facilities is personalised and holistic. Here, children and adolescents with chronic illness or mental health conditions receive targeted support in dealing with their illness. The aim throughout is to sustainably improve quality of life, and participation in education and in everyday life.

Unfortunately, children from families with a migration background rarely take up the opportunity of accessing medical rehabilitation. The MiMi-Reha-Kids project of the Ethno-Medical Centre Inc. (Ethno-Medizinisches Zentrum e.V.) and Lübeck University (Unversität zu Lübeck) responds by aiming to lower the barriers to accessing medical rehabilitation that these children and adolescents experience. This guide has been developed as part of this endeavour. It aims to inform parents and young people about the medical rehabilitation services available to them, assuage any reservations they may have, and draw attention to the opportunities that treatment at a rehabilitation facility has to offer. Moreover, it contains practical tips and assistance with lodging an application. I sincerely hope that many more families with a migration background will learn about medical rehabilitation services, and that their uptake will increase.

I hereby thank all who have contributed to the development of this guide.

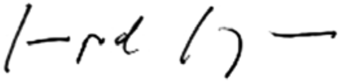

Dr. Ingrid Künzler,
Chairperson of the Executive, German Pension Insurance, Northern Regional Fund (DRV Nord)

The most important facts first

- If your child has a ***chronic**** **illness,** medical rehabilitation for children and adolescents can **help a lot to improve health.** At rehabilitation facilities, children and adolescents receive personalised and holistic treatment. It has a focus on learning how to deal with the illness. This can significantly **improve your child's quality of life.**

- Rehabilitation facilities treat a wide range of illnesses. The majority of patients seek treatment at a rehabilitation facility because of a ***mental health condition.*** The next most common health problems include **obesity, asthma, neurodermatitis, and spinal and back problems.**

- Rehabilitation facilities are staffed by **multidisciplinary teams.** This means that, apart from doctors and nursing staff, a whole range of other specially trained therapists also work there. Together, they can offer each patient their own **personalised and varied treatment plan.** Different services (e.g. strength training or dance therapy) can help improve the overall state of health. They also include learning to deal with the illness in everyday life.

** Terms printed in italics are explained in the glossary.*

- Patients spend about four weeks **at the health facility and also receive their meals there.** School-age children also continue to attend **school lessons** at the facility. Apart from the therapeutic interventions, group and **leisure activities** are also offered. Depending on the facility, there may be an adventure or high ropes course, a swimming pool, or basketball courts. This way they also get to know other affected children, which is often very beneficial. Children up to the age of 12 years are usually **accompanied by one of the parents.** Older children may also be accompanied if necessary, with costs fully covered.

- Several institutions within the German social and health care systems finance rehabilitation for children and adolescents. The two most important are **German Pension Insurance (Deutsche Rentenversicherung, DRV)** and **Statutory Health Insurance (Gesetzliche Krankenversicherung, GK).** In this guide, we explain in detail how to **lodge an application with German Pension Insurance (Deutsche Rentenversicherung, DRV).** Important to know: If you have a child with a *chronic* illness and think that treatment at a rehabilitation facility might be sensible, lodging an application is worthwhile in most cases. If the agency you lodged the application with is not responsible in your case, it is obliged to forward the application to the responsible agency. This means that you have a **good chance that your application will be successful**, and that your child will have the opportunity to make a sustainable improvement to its health status and quality of life through treatment at a rehabilitation facility.

Contents

Introduction
What kinds of information does this guide offer? 9

1 The basics
What is medical rehabilitation for children and adolescents? 10

2 Health conditions
Which types of illness can be treated at a rehabilitation facility? 12

3 At the rehabilitation facility
What actually happens there? 22

4 Rights and entitlements
Criteria for the costs of rehabilitation
for children and adolescents to be covered 26

5 Lodging the application
How does it work? 30

Appendix
Case studies 36
FAQ 40
Services directory 43
Glossary 50

Introduction

What kinds of information does this guide offer?

Children who are ill have a right to the best possible medical care. The German health system offers comprehensive support, but can also be difficult to navigate. Some treatment options are not sufficiently well known. With this guide, we would like to provide interested parents with fundamental information about medical rehabilitation. Medical rehabilitation is a very good option for children and adolescents with chronic illness to improve their state of health and quality of life for the long term.

In **Chapter 1,** we explain what medical rehabilitation for children and adolescents means.

In **Chapter 2,** we discuss the types of illness that can be treated at rehabilitation facilities.

In **Chapter 3,** we provide practical information about staying at a rehabilitation facility and about its daily routines.

In **Chapter 4,** we look at the various agencies with whom applications for medical rehabilitation for children and adolescents can be lodged. In this guide, we focus on agencies that are part of German Pension Insurance (DRV).

In **Chapter 5**, we go through the application form for medical rehabilitation for children and adolescents step by step, and explain what to keep in mind when lodging an application with one of German Pension Insurance's (DRV) individual pension funds.

In the appendix, you will find fictional case studies, a list of frequently asked questions and answers (FAQ), a services directory with contact persons, service agencies and their contact details, as well as a glossary of important terms on the topic of medical rehabilitation for children and adolescents.

We hope that this guide will assist interested parents of children with chronic illness, and encourage them to take advantage of medical rehabilitation services for their children. In order to reach as many people as possible, this guide is available in nine languages in addition to German.

1 The basics

What is medical rehabilitation for children and adolescents?

An illness is called *chronic* if it lasts for a long time and can't be completely cured. *Chronic* illness can severely restrict the lives of children and adolescents. It may affect home life, but also school, vocational training, employment or study.

Medical rehabilitation (often shortened to 'Reha' in German) can help in these cases. Its aim is to improve patients' *mental* health, so that they may lead the best life they can, despite their illness. The treatment provided during medical rehabilitation is more varied than that offered e.g. at a hospital. During medical rehabilitation, patients learn to better understand their illness, and to practice how to deal with it better in everyday life. You may have heard people use the German term *Kur,* a now obsolete word that is still sometimes used colloquially for medical rehabilitation in German-speaking countries.

Typical health conditions treated in rehabilitation facilities include *mental* health conditions, especially ADHD, *depression,* severe stress reactions, adjustment disorders and developmental language disorder, as well as asthma, obesity (severe overweight), skin diseases, deformities of the spine and back, and cancer. However, medical rehabilitation may also be the right course of action for other *chronic* conditions in order to improve the general state of health, and to be able to enjoy life despite the illness.

Germany has a multitude of *inpatient* rehabilitation facilities that cater to children and adolescents in particular. These facilities offer age-appropriate accommodation, and patients stay for four weeks in most cases. School lessons are also provided to cover the most important subjects of the curriculum. Children under the age of 12 years and children with special needs are accompanied by a caregiver (one of the parents in most cases). The accompanying person also receives

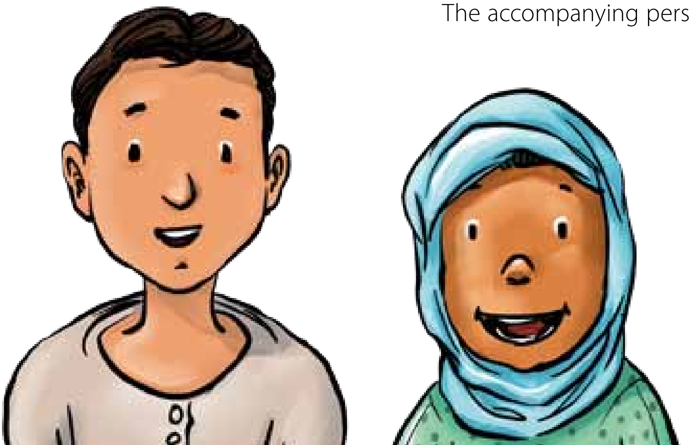

accommodation and meals at the facility, but not medical treatment. Siblings up to the age of 12 years may also accompany the patient. This type of medical rehabilitation is called *inpatient* rehabilitation (stationäre Reha). In most cases, it will not be taking place near your place of residence.

In addition, health care facilities in some locations have recently begun to offer the option of *outpatient* medical rehabilitation (ambulante medizinische Rehabilitation). This means that the rehabilitation facility is located near the patient's place of residence. Children and adolescents then attend the facility for several hours during the day, but spend their nights at home. They can then keep attending their usual school, traineeship or apprenticeship, workplace or university. There aren't yet many *outpatient* rehabilitation facilities for children and adolescents. But more will be established in the future.

German Pension Insurance (Deutsche Rentenversicherung, DRV), through its various pension funds, pays for medical rehabilitation for children and adolescents because treating illness early can prevent severe damage to health later.

However, other agencies also pay for medical rehabilitation in certain cases. For administrative purposes, agencies that cover the costs of such health services are called 'Kostenträger'. Apart from German Pension Insurance (DRV), the most important other 'Kostenträger' agency for medical rehabilitation is Statutory Health Insurance (Gesetzliche Krankenversicherung), through its various insurance funds. In Chapter 5, we explain the different cost-covering agencies (Kostenträger) and their criteria for financing medical rehabilitation for children and adolescents.

2 Health conditions

Which types of illness can be treated at a rehabilitation facility?

According to parents, almost every sixth underage young person in Germany (16.2%) suffers from a *chronic* illness. This is equivalent to more than two million children and adolescents. *Chronic* diseases are long-term health conditions that often require lifelong medical care. Normally, local paediatricians (Kinderärzt*innen) and medical specialists (Fachärzt*innen) treat *chronic* illness. If this treatment is not sufficient, medical rehabilitation for children and adolescents is an additional option. The following is a brief overview of some of the conditions that are often treated through medical rehabilitation. However, these are only examples. Medical rehabilitation can also be useful in cases of anorexia, cardiovascular disease, neurological diseases and other conditions. If you are unsure whether medical rehabilitation is a viable option based on your child's *chronic* condition, it is best if you talk to your paediatrician or your general practitioner.

Mental health conditions

Mental illness is very stressful for children and adolescents, and for their families. Often, these kinds of illness don't just disappear by themselves, and are still present when the child reaches adulthood. Rehabilitation facilities treat many different types of *mental* health conditions, from ADHD and *addictions* to post-traumatic stress disorder.

ADHD

Attention Deficit Hyperactive Disorder (ADHD) is one of the most common *mental* health disorders in childhood and adolescence. Children and adolescents who suffer from ADHD are often inattentive and find it hard to control their own behaviour. At the same time, they have a strong urge to move and are very active, which is why children with this condition are often called 'hyperactive'. At school, these children often lack concentration, which leads to learning difficulties. Conflict with other children and problems with going to sleep are also common. It often happens that the children's hyperactive behaviour is blamed on bad parenting. However, ADHD has nothing to do with parenting. In Germany, about 4.4% of children and adolescents between the ages of 3 and 17 years are *diagnosed* with ADHD. However, boys receive an ADHD *diagnosis* more than twice as frequently as girls. This means that ADHD is not rare, but affects many children and adolescents in Germany.

The following methods are typically used at rehabilitation facilities to treat ADHD:

- Family and behavioural therapy
- Illness-specific training courses for patients and their relatives
- Counselling sessions for relatives
- *Psychological* interventions and art therapy
- Self-awareness and life skills training
- Relaxation training
- Social work, school and social integration

DEPRESSION

Not only adults suffer from *depression*, this mood disorder also occurs regularly among children and adolescents. *Depression* manifests as persistent sadness, listlessness and negative thoughts. Children who suffer from *depression* are often sad, even when, from an outside perspective, there is no apparent reason. Other people often can't understand why the person is having such a bad time. In contrast to adolescents and adults, *depression* in younger children may manifest more strongly as physical symptoms such as loss of appetite, sleep disorders and frequent stomach ache. *Depression* is normally *diagnosed* when these signs persist for 14 days or longer. It is assumed that various causes, such as a genetic predisposition and traumatic experiences, contribute to *depression*. However, this doesn't mean that a terrible experience affects everyone in the same way. Some people can deal with traumatic experiences more easily than others. This means that a terrible experience may or may not be contributing to the *depression*.

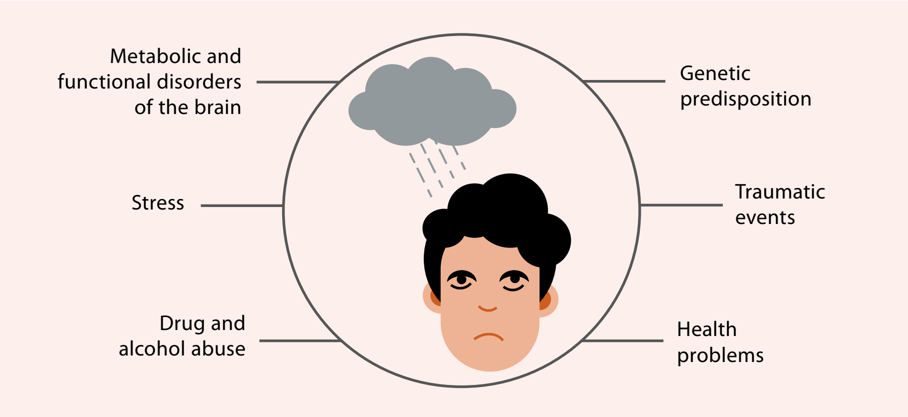

Fig. 1: Causes of depression

Depression is not a sign of weakness, and it can affect anyone. In Germany alone, more than 100,000 children and adolescents under the age of 18 years were *diagnosed* with *depression* in 2017.

The following methods are typically used at rehabilitation facilities to treat *depression*:

- *Psychotherapy*
- Art therapy
- Illness-specific training courses for patients and their relatives
- Counselling sessions for relatives
- Self-awareness and life skills training
- Movement therapy/movement games

POST-TRAUMATIC STRESS DISORDER

Post-traumatic stress disorder (PTSD) occurs in children and adolescents as it does in adults. PTSD is a *psychological* reaction to a stressful event, and is accompanied by *psychological* and *psychosomatic* symptoms. Stressful events may include, for example, an accident, a violent crime or a natural disaster. But not every terrible event necessarily results in PTSD. People who suffer from PTSD often experience recurrent unwanted memories of the event, called flashbacks. Some children keep re-enacting the event through play. It's also possible that the child hardly remembers the event at all and refuses to talk about it. Some children also react by 'unlearning' already acquired skills – they may, for example, begin to wet themselves again. Sleep problems, tantrums, difficulties concentrating and an unusual jumpiness can also be signs of PTSD. The prevalence of PTSD is related to the occurrence of traumatic events. PTSD is therefore likely to occur more often in crisis regions, e.g. war zones or natural disaster areas. Refugee children are especially affected by PTSD because of the sometimes traumatic events they experience on their journey. In Germany, about every fifth adolescent between 12 and 17 years of age has had a traumatic experience, but only a few of them show signs of PTSD (1.6%). Girls are significantly more frequently affected by PTSD because they experience severe trauma – such as repeated sexual violence – more often than boys do. Adolescents with PTSD often have additional health conditions such as *depression* or substance abuse.

The following methods are typically used at rehabilitation facilities to treat PTSD:

- *Psychotherapy*
- Art therapy
- Illness-specific training courses for patients and their relatives
- Counselling sessions for relatives
- Self-awareness and life skills training
- Social work, school and social integration

Developmental language disorder

The ability to use language to communicate distinguishes humans from other life forms. For most people, using language is an important means of participating in society. It enables them to acquire knowledge, and to exchange information, feelings and ideas.

If a child performs markedly weaker than its peers when it comes to pronunciation, grammatical rules, vocabulary or applying communication skills in practice (e.g. understanding language in a given situation), it may be affected by developmental language disorder.

Language problems may have various causes. They may be the result of hearing loss, or they may occur as part of a genetic condition such as Down syndrome. An unfavourable learning environment, such as a lack of language modelling by other people, can also have an impact on language development.

Developmental language disorder can cause far-reaching social and *psychological* problems, and can permanently limit the affected children's educational opportunities and quality of life. If language development appears abnormal, it is important to access therapeutic assistance early in order to allow the affected child to develop in a way that is appropriate for its age.

Few statistics are available on the prevalence of developmental language disorder in children. In English-speaking countries, the prevalence of developmental language disorder in kindergarten children is estimated at around 6 to 8%. Boys are affected more frequently than girls.

The following methods are typically used at rehabilitation facilities to treat developmental language disorder:

- Speech therapy
- *Physiotherapy* and *occupational therapy*
- Music therapy
- Illness-specific training courses for patients and their relatives
- Self-awareness and life skills training
- Social work, school and social integration

Obesity and overeating

Obesity is the excessive accumulation of body fat. The development of obesity cannot be attributed to a single cause. Reasons for the development of obesity are, for example, a genetic predisposition, or a lifestyle with little exercise, high media consumption and a diet high in sugar and fat. Many children and adolescents who are very overweight suffer from back and joint pain, which means that they exercise less and less. Children with obesity also tend to experience exclusion and degrading comments from their peers. Apart from these immediate problems, these children and adolescents are at a higher risk of additional *chronic* conditions such as diabetes and high blood pressure (hypertension) later in life (Fig. 2). Because some health problems only emerge later in life, this risk is easily underestimated. That's why it's important to seek help early.

In Germany, about 15.5% of all boys and girls between the ages of 3 and 17 years are overweight, and around 5.9% of all children and adolescents in this age group are severely overweight, i.e. obese.

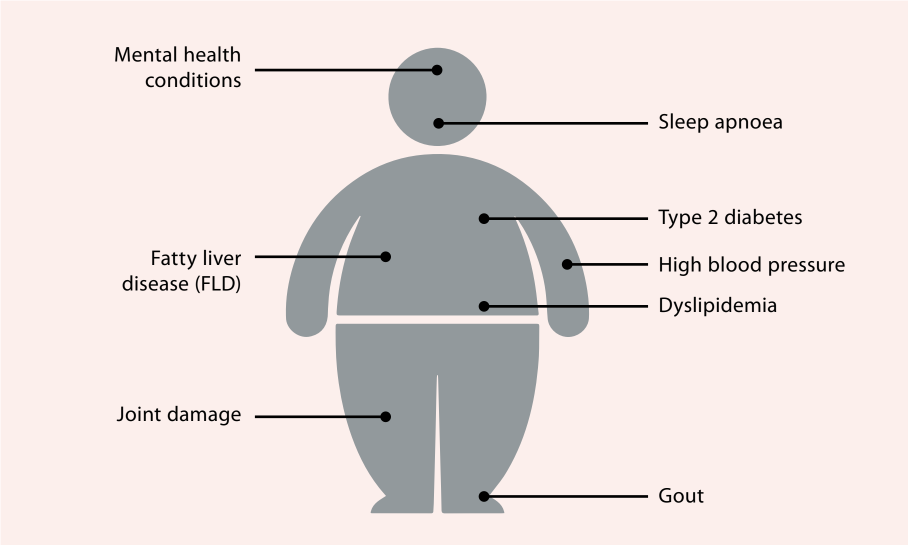

Fig. 2: Health effects of obesity

The following methods are typically used at rehabilitation facilities to treat obesity:

- Personalised dietary advice
- Cooking classes for parents and children
- Food shopping skills for adolescents and parents
- Behavioural therapy
- Movement therapy/movement games
- Self-awareness and life skills training
- Illness-specific training courses for patients and their relatives
- Vocational orientation and integration
- Endurance training
- Swimming

Asthma

Asthma is a condition where the airways become *chronically* inflamed. Children and adolescents with asthma suffer from shortness of breath, cough and hypersensitive bronchi. People with asthma also experience a whistling or humming noise while breathing, called 'wheezing'. Many children and adolescents with asthma also feel like their chest is constricted. Symptoms are sometimes stronger, sometimes weaker, and may vary in intensity over time. Asthma can severely affect quality of life. Because they cannot participate in activities to the same extent as their peers, children and adolescents with asthma can feel excluded and may withdraw. With the right medical support, asthma can be controlled successfully, and quality of life can improve significantly for the affected children. This requires that the condition is detected and treated as early as possible. In Germany, about 4.0% of those between the ages of 3 and 17 years have been *diagnosed* with asthma. At 4.2%, boys are affected slightly more often than girls at 3.1%. This makes asthma one of the most prevalent *chronic* conditions in childhood and adolescence.

The following methods are typically used at rehabilitation facilities to treat asthma:

- Breathing exercises
- How to use inhaler ('puffer') and peak flow meter
- Movement therapy
- Illness-specific training courses for patients and their relatives
- *Psychological* interventions and art therapy

Neurodermatitis

The skin is the largest human organ. Its many sensory receptors make it our main communication link with our environment. It also regulates body temperature and fluid balance. At the same time – because of its position at the outer boundary of the body – it is our first line of defence against foreign substances, and is therefore constantly exposed to environmental influences, which can potentially damage it.

Rehabilitation facilities for children and adolescents treat many skin conditions such as neurodermatitis, psoriasis, acne and hives (urticaria). The most frequently treated skin condition is neurodermatitis. The causes of neurodermatitis are still not fully known. Triggers of the disease can include exposure to environmental toxins such as tobacco smoke, cosmetics, detergents and allergens. *Psychological* and other types of stress can also contribute to its development. People with neurodermatitis often have dry skin with reddened and inflamed patches called eczema. These areas can be very itchy. Nodules and pustules may also develop in the affected areas. Depending on age, neurodermatitis occurs in different areas of the body. Infants mostly develop itchy, reddish patches of skin on the cheeks and head. In children, typically affected areas are the insides of the elbows, the back of the knees, and the wrists (Fig. 3). The itching is usually worse in the evening and at night than during the day, and leads to poor sleep. For this reason, those affected

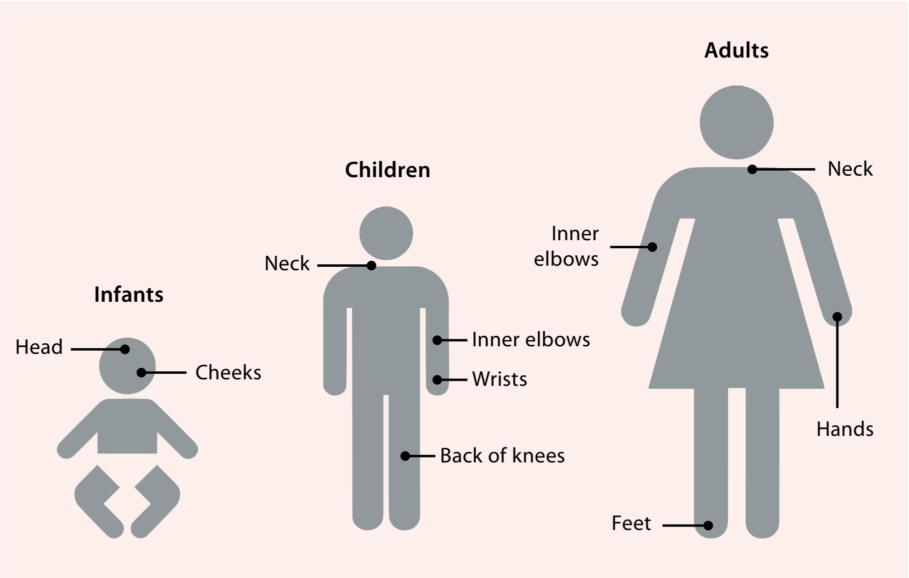

Fig. 3: Body parts frequently affected by neurodermatitis by life stage

may feel tired and lack concentration during the day. In most cases, neurodermatitis first manifests even before a child's second birthday. Overall, around 12.8% of children and adolescents in Germany have neurodermatitis. Girls are affected slightly more frequently than boys.

The following methods are typically used at rehabilitation facilities to treat neurodermatitis:

- Learning skin care/ personal hygiene skills
- Relaxation techniques
- Self-awareness and life skills training
- Illness-specific training courses for patients and their relatives
- Counselling for relatives

Spinal and back problems

In adults, malformations of the spine and the back are among the most common reasons for accessing medical rehabilitation. However, for spinal and back problems, medical rehabilitation can also be useful in childhood and adolescence. Apart from the classic hollow back (lordosis) and hunched back (kyphosis) posture problems, many different back and spinal conditions are treated in medical rehabilitation for children and adolescents. Malformations can be congenital, but often they only develop over time. Affected children and adolescents tend to suffer from back pain, headaches and neck pain. Pain in the knees and ankles can also result from these conditions. Deformities of the spine and back in childhood and adolescence may lead to those affected being permanently restricted in their mobility and activity levels in adulthood. This is also related to the fact that deformities represent an increased risk of secondary health effects such as herniated disks. It is therefore important that parents work together with the treating doctors to reduce this risk. In Germany, about 4.9% of girls between the ages of 3 and 10 years suffer from back pain, with a similar rate of 4.5% affected among boys. It is a known fact that the prevalence of back pain increases with age, as muscles, joints and bones are placed under more and more strain. Accordingly, as much as 28.3% of girls and 19.9% of boys among those aged 11 to 17 years report back pain.

The following methods are typically used at rehabilitation facilities to treat back and spinal problems:

- Movement therapy
- *Physiotherapy* (e.g. neurophysiological therapy according to Bobath, hydrotherapy, therapeutic indoor rock climbing, breathing exercises)
- *Occupational therapy*
- Illness-specific training courses for patients and their relatives
- Relaxation techniques
- *Induction to medical aids*
- How-to-walk training
- Swimming

Cancer

Cancer is the malignant development of certain body cells. In contrast to the regulated growth of healthy cells, cancer cells grow out of control, don't serve any specific function and destroy the surrounding tissue. Children and adolescents who suffer from cancer are exposed to a great deal of physical and *mental* stress. Childhood cancer generally also presents a great challenge to parents, siblings and other loved ones, a challenge that requires a lot of strength. It is not uncommon for relatives to experience severe stress because of the disease as well, and to need support. Annually over the past few years, an average of 1774 cases of cancer have been reported in children under the age of 15 years, and 365 cases in children and adolescents aged between 15 and 17 years. Blood cancer is one of the most common cancers in children and adolescents. Blood cancer develops in the bone marrow. It means that the usual multiplication of immature white blood cells in this body tissue becomes unregulated and gets out of control. Boys are slightly more frequently affected by blood cancer than girls. Today, the chances of recovery for children and adolescents are good. In general, 82% of those under the age of 15 years survive cancer by at least 15 years. For blood cancers, the 15-year survival rate is 90% for this age group. Medical rehabilitation can help children and adolescents to regain their strength after such a stressful time, thus reducing the effects of the illness.

The following methods are typically used at rehabilitation facilities to treat cancer patients:

- Illness-specific training courses for patients and their relatives
- Counselling sessions for relatives
- *Psychological* interventions and art therapy
- Self-awareness and life skills training
- Social work, school and social integration

Effectiveness of medical rehabilitation

Current research shows that many of the medical and therapeutic services provided during rehabilitation can improve the health and quality of life of children and adolescents with *chronic* illness. Interventions whose positive effects have been confirmed by studies include illness-specific training for patients and their children in cases of ADHD, asthma, neurodermatitis and overweight, as well as movement therapy in cases of asthma, back problems and overweight. In order to ensure the best possible treatment for children and adolescents in rehabilitation facilities, German Pension Insurance (Deutsche Rentenversicherung) also uses a range of quality assurance tools. Therapeutic standards for rehabilitation (Reha-Therapiestandards), for example, ensure that treatment is provided on the basis of scientific evidence. Regular patient surveys ensure that the perspective of those who are affected is also considered in the continuous improvement of medical rehabilitation. Medical rehabilitation can therefore make an important contribution to improving the health and quality of life of children and adolescents. This means that it represents an important component of the health care provided to children and adolescents in Germany from a scientific perspective as well.

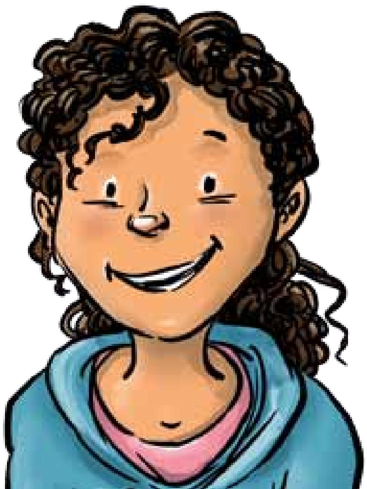

3 At the rehabilitation facility

What actually happens there?

Germany has many facilities specialising in child and adolescent rehabilitation, which treat a wide range of conditions. Most facilities are located in the north of Germany – on the North Sea and Baltic Sea coasts – and in southern Germany's alpine and prealpine regions.

In most cases, children and adolescents spend around one month at the facility. They receive a comprehensive treatment plan (Therapieplan), which does not solely focus on the chronic illness itself, but takes into account the entire life situation of the children and their relatives. In order to ensure optimal care, the children and adolescents are looked after by a team in which doctors, nurses, educators and other professionals work together. For example, treating an adolescent with obesity may, apart from sports and movement sessions, also comprise psychotherapeutic interventions, cooking classes, special training courses for family members, as well as conversations about the young person's educational and employment outlook. Treatment interventions and exercises take place individually and in groups. Groups are composed so that the children and adolescents are in contact with their peers, with whom they can exchange experiences during medical rehabilitation. To ensure that the children and adolescents don't fall behind in their schoolwork during this period, regular lessons are also held at the facility. This broad rehabilitation treatment spectrum ensures that each child receives exactly the support it needs.

The duration of rehabilitation

Rehabilitation for children and adolescents takes at least four weeks and can be extended if required. The actual duration is individually tailored to your child's *chronic* health condition and to your personal situation. It is negotiated through close communication between you and the rehabilitation facility.

Packing list

Before you travel, we advise that you discuss with the rehabilitation facility what your child should bring. Normally, patients will need their health insurance card (and further documents such as ID card if required), medical documentation (e.g. *medical reports*, x-ray images, any relevant emergency documents), regularly taken medications, individual medical aids (e.g. glasses, hearing aid etc.), sportswear and swimwear, personal hygiene products, weatherproof clothing, shoes and personal items. It is, in principle, the same as packing for a holiday trip. In order to estimate the right amount of clothing to take,

you can ask whether laundry facilities will be available.

Travel

You must must make your own arrangements for travel to the facility, by car or by public transport. Your statutory pension fund (Rentenversicherung) will reimburse you at the rate of the cost of public transport. For car travel, the reimbursement rate is set at 20 cents per kilometre, with an upper limit of 130 euros including related fees, such as car parking.

Accommodation

Smaller children share accommodation with their accompanying adult, in most cases one of the parents. Unaccompanied older children and adolescents mostly share accommodation in single-sex groups with other children of a similar age and with similar health problems. This is an important aspect of rehabilitation. In everyday life, many children and adolescents with *chronic* illness struggle with the fact that they often find themselves alone with their situation. Talking with children and adolescents in similar situations can provide renewed strength, and help them to handle the illness better. You can discuss all questions regarding accommodation and daily routines, e.g. your child's meals, sporting activities or religious observance, with the staff at the facility in advance. The staff will accommodate your wishes as much as possible.

Treatment plan

After arriving at the rehabilitation facility, the treating doctor will have a detailed conversation with you and your child in order to get a more accurate picture of the health situation. If you need it, German Pension Insurance (DRV) will pay for an interpreter to assist with this initial consultation. However, this only applies to the initial consultation; you will not be provided with language assistance for the other components of rehabilitation.

The initial consultation also includes a physical examination of your child. You can ask in advance what exactly this will include, so that you can explain it to your child and alleviate any fears.

After the consultation and the examination, the rehabilitation team puts together an appropriate treatment plan (Therapieplan). The treatment plan is similar to a school timetable. It lists all the rehabilitation interventions (Reha-Maßnahmen), which is the name used for all the different types of treatment, therapy sessions and exercises that patients receive. This ensures that all staff members (doctors, therapists, care personnel and others) who are responsible for your child, know the treatment goals and can work towards them collaboratively. In addition, you as a parent or accompanying adult will be involved from the beginning, so that you can continue with the activities when everyday life resumes at home later.

Daily routine

During the day, your child will participate in scheduled treatment interventions, lessons, meals and leisure activities. Treatment interventions include movement exercises (*occupational therapy, physiotherapy,* sports), relaxation training, creative methods (art, music), *psychological* and social interventions (individual or group therapy and counselling sessions). There may also be time to learn how to use potential medical aids, e.g. a prosthesis (*induction to medical aids*, called Hilfsmitteltraining in German). Each intervention is scheduled several times per week, and interventions alternate over the course of each day. You can find more detailed descriptions of treatment interventions in the information boxes in Chapter 2. Moreover, patients – and accompanying adults if required – will attend training sessions where they receive information about medications and other forms of treatment for the relevant *chronic* illness. School lessons are held in one block, mostly in the mornings, but extending to the afternoon if required. Outdoor education and leisure activities are closely connected to treatment interventions. How to deal with your own limitations in a group is explored during joint activities. Social and motor skills are also practiced as part of these activities.

During the course of rehabilitation, the team keeps reviewing the treatment plan and adjusts it if necessary. Before the end of the rehabilitation period, the doctor will conduct another examination and detailed consultation with you and your child. It includes a discussion about how the rehabilitation went, as well as recommendations to follow when you return home. The aim is to support patients and their families with resuming their everyday life at home.

Accompanying adults

For children up to the age of 12 years, you can attend rehabilitation together with your child. Where medically indicated, this may also apply to older children.
In many cases, it may also be beneficial for an adolescent to stay at the rehabilitation facility unaccompanied.

School lessons

If your child is at school, it will attend weekday lessons throughout the rehabilitation period. These are closely coordinated with the teachers at your usual school. Moreover, test papers and exams can also be taken at the facility so that patients don't fall behind their classmates at home. Class sizes are often smaller than at their usual school. This enables the teaching staff to provide individual support. It helps to recognise problems with schoolwork more quickly, and overcome them where possible. After leaving the rehabilitation facility, a final report is sent to the school at home, including information about the rehabilitation period and any recommendations.

A special case: Family-oriented rehabilitation

In cases of very severe illness, such as cancer or cystic fibrosis, the German health system offers the option of Family-Oriented Rehabilitation (FOR, familienorientierte Rehabilitation). FOR means that apart from the child who has the illness, parents (or other persons who have custody of the child) and siblings are also admitted to the rehabilitation facility. Family-Oriented Rehabilitation (FOR) becomes an option when the family's everyday life is severely affected by the child's illness, and if it seems necessary for rehabilitation to be successful that parents and siblings accompany the child. Apart from treating the child who has the *chronic* illness, the rehabilitation facility also provides personalised therapeutic care for the other family members. The aim is to stabilise and empower the whole family.

Outpatient services

In future, more and more *outpatient* services are planned for child and adolescent rehabilitation. Patients will then spend several hours at their rehabilitation facility during the day. However, so far there are only two such facilities in Germany (Cottbus and Cologne). There will be more and more such services in the future. *Outpatient* services are mainly suited to health conditions and problems that can chiefly be improved through regular treatment that takes place within the person's usual social environment. Examples include overweight, and psychosocial problems such as bullying, or parenting issues.

Distinction from parent-child rehabilitation (Eltern-Kind Kur)

In medical rehabilitation for children and adolescents, the focus is solely on your child who has a *chronic* illness. As an accompanying adult, you receive training and guidance to learn and improve how you are dealing with the illness. Rehabilitation for children and adolescents should not be confused with *inpatient* parent-child rehabilitation (stationäre Eltern-Kind *Kur)*. Parent-child rehabilitation is about you as a mother or father, in cases where your own health is compromised or at risk. Reasons include stress-related exhaustion, family separations and parenting problems. Children up to the age of 12 years (in exceptional circumstances up to 14 years) may accompany you to the rehabilitation facility.

4 Rights and entitlements

Criteria for the costs of rehabilitation for children and adolescents to be covered

Cost-covering agencies

In Germany, the costs of medical rehabilitation may be covered by a range of statutory and other agencies. In fulfilling this function, they are called Kostenträger or Rehabilitationsträger ('cost carriers' or 'rehabilitation carriers'), and it is with one of them that you must lodge your application for medical rehabilitation. In this guide, we focus on German Pension Insurance (Deutsche Rentenversicherung) as one of the statutory agencies that can cover the costs of medical rehabilitation. Additional agencies that may cover rehabilitation costs are briefly introduced in the section titled 'Other cost-covering agencies'. Importantly, you can lodge an application for medical rehabilitation with any of the agencies that have this function of 'Kostenträger' ('cost carrier'). If the agency with whom an application for medical rehabilitation has been lodged is not the one responsible for covering the costs in that particular instance, the agency is obliged to forward the application to another cost-covering agency they consider more appropriate.

GERMAN PENSION INSURANCE (DEUTSCHE RENTENVERSICHERUNG, DRV) AS COST-COVERING AGENCY

Medical rehabilitation for children and adolescents with particular types of *chronic* illness is a prescribed benefit of German Pension Insurance (DRV) as a statutory body. This means that German Pension Insurance (DRV) is mandated by law to cover this type of costs. As long as an applicant meets the personal requirements and the requirements relating to insurance law, the application must be approved.

Personal requirements include:

- A *positive rehabilitation prognosis:* there must be a good chance that medical rehabilitation can help your child. If a child is so severely ill or disabled that it will probably never enter paid employment, the application should be lodged with another cost-covering agency, e.g. statutory health insurance (Krankenkasse).
- *Rehabilitation need:* your child must be suffering from a *chronic* illness that is limiting or endangering the child's health. If the chronic condition is clearly limiting your child's social life, and may be putting its later *earning capacity* at risk, *rehabilitation need* is established.
- *Rehabilitation capacity:* your child must be physically, psychologically and socially capable of participating in medical rehabilitation.

More on this topic in Chapter 5.

The **requirements relating to insurance law** are about the *social insurance* status of the person who has custody, and who is lodging the application on behalf of the child or adolescent. If this person is employed or has been employed until recently, is in vocational training (apprenticeship or traineeship) or in receipt of a state pension, or if the child has lost one or both parents, the requirements relating to insurance law are usually met. As described above, you can simply lodge the application with German Pension Insurance (DRV), even if you are not entirely certain that all requirements have been met. If German Pension Insurance (DRV) staff determine that German Pension Insurance (DRV) is not the responsible cost-covering agency in this instance, they will pass the application on to one of the other cost-covering agencies.

Other cost-covering agencies

STATUTORY HEALTH INSURANCE

Statutory health insurance funds also cover the costs of medical rehabilitation treatment for children and adolescents in certain cases. All children and adolescents covered by statutory health insurance are entitled to this benefit.

PRIVATE HEALTH INSURANCE

Children with private health insurance are not always entitled to have the costs of medical rehabilitation covered by their insurance fund. Whether medical rehabilitation is covered depends on the individual insurance policy. It may either cover medical rehabilitation for children and adolescents, or it may not.

HEALTH ALLOWANCE (BEIHILFE)

The health allowance (Beihilfe) is a separate scheme covering health care provision for German public officials. It can also cover the costs of medical rehabilitation for their children.

STATUTORY ACCIDENT INSURANCE

Statutory accident insurance covers the costs of rehabilitation treatment for children and adolescents that have become necessary as a result of an accident at a day care centre (Kita), at school, at the workplace, or on the way to or from one of these places. In contrast to statutory pension insurance and statutory health insurance, statutory accident insurance takes action as soon as it has been notified of an accident. This means that the insured person doesn't have to lodge an application.

YOUTH ASSISTANCE (JUGENDHILFE)

Offices for youth affairs (Jugendämter) only pay for medical rehabilitation in case of *mental* disability. *Mental* disabilities refer to *mental* health conditions such as personality and behavioural disorders, schizophrenia, persistent *depression* and anxiety disorders. Offices for youth affairs (Jugendämter) cover the costs of rehabilitation for all children and adolescents, regardless of whether the person who has custody is covered by statutory pension or health insurance.

Refugee children – a special scenario

During the first 18 months after arriving in Germany, children who are refugees receive benefits according to the Asylum Seeker Benefits Act (Asylbewerberleistungsgesetz), which does not cover medical rehabilitation. During these 18 months, they are also not covered by statutory health insurance. Most do not meet the insurance law requirements of German Pension Insurance (DRV) during this period either.

However, in cases of *mental* or *psychiatric* disability, it is possible to lodge an application through youth assistance (Jugendhilfe) during this period, as long as those affected are living in Germany legally or on the basis of a lawful concession to stay (Duldung). This includes asylum seekers. Refugee children are exposed to particular types of *psychological* stress. For *mental* health conditions, and for PTSD in particular, medical rehabilitation can be a very good treatment option.

Other legal provisions

AGE LIMIT

In principle, adolescents up to the age of 18 years can apply for medical rehabilitation. In some cases, young adults up to 27 years of age can access medical rehabilitation for children and adolescents if they are serving in one of the German government-sponsored volunteer and civil engagement schemes for young adults. These include the voluntary social or ecological service (Freiwilliges Soziales oder Ökologisches Jahr) and the Federal Voluntary Service (Bundesfreiwilligendienst). People with a disability who are unable to support themselves independently can also access medical rehabilitation for children and adolescents up to the age of 27 years. If they have already made sufficient statutory pension contributions of their own (six months during the last two years), they can apply for rehabilitation through their statutory pension fund (Rentenversicherung). In this case, however, this form of rehabilitation is adult medical rehabilitation, not medical rehabilitation for children and adolescents. These two types of rehabilitation services differ in a number of ways. Older adolescents who are eligible for both types of rehabilitation are strongly encouraged to seek advice, e.g. at one of the German Pension Insurance service points.

COSTS

You will not incur any costs when your child attends medical rehabilitation. In contrast to adult medical rehabilitation, medical rehabilitation for children and adolescents through German Pension Insurance (DRV) is free from co-payments. All costs are covered by German Pension Insurance (DRV).

ACCOMPANYING PARENTS AND OTHER CAREGIVERS

Children up to the age of 12 years may be accompanied by one other person (one of the parents in most cases). If medically indicated, older children may also be accompanied. The accompanying person does not receive medical treatment while staying at the facility. However, they do receive accommodation and meals. Siblings may also be brought along if they cannot otherwise be cared for. German Pension Insurance (Deutsche Rentenversicherung) covers the costs of accommodation and meals, as well as travel between home and the facility. It is also possible to receive reimbursement for the costs of household assistance. In addition, the accompanying adult can apply for compensation of any lost earnings. Livelihood support benefits such as unemployment benefit II (Arbeitslosengeld II, colloquially called 'Hartz 4'), continue to be paid while the recipient stays at the rehabilitation facility. Moreover, several accompanying adults (e.g. both parents) can share the rehabilitation period. For the most severe types of *chronic* illness, family-oriented rehabilitation (FOR) may also be an option. With this form of rehabilitation, the whole family receives treatment. You can find more detailed information about FOR in Chapter 3.

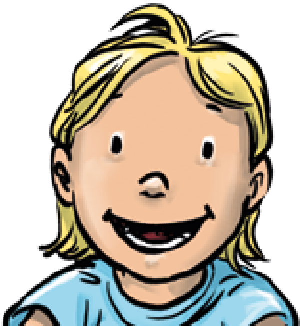

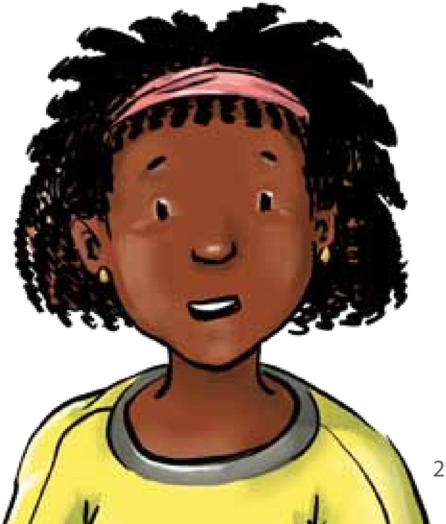

5 Lodging the application

How does it work?

In this chapter, we explain what to pay attention to when lodging an application with German Pension Insurance (Deutsche Rentenversicherung, DRV) and how to fill in the form. You can request the application forms from your own statutory pension fund. The application form can also be downloaded as a PDF, which is part of the application package for medical rehabilitation for children and adolescents, on the German Pension Insurance website (www.deutsche-rentenversicherung.de/SharedDocs/Formulare/DE/Formularpakete/01_versicherte/reha/_DRV_Paket_Rehabilitation_Kinderrehabilitation.html).

Information about lodging applications with statutory health insurance funds (gesetzliche Krankenkassen), private health insurance companies, statutory accident insurance (gesetzliche Unfallversicherung), health allowance (Beihilfe) or offices for youth affairs (Jugendämter) can be obtained from the respective cost-covering agency. For an explanation of the different criteria for cost coverage, see Chapter 4.

You can ask your paediatrician for assistance with lodging an application at any time.

Step-by-step application

1. DOCTOR'S RECOMMENDATION

First, the parents or the adolescent must talk to the treating paediatrician or general practitioner. Crucial for application and approval is the *medical report (ärztlicher Befundbericht),* using form G0612. In this report, the treating doctor sets out the patient's medical history, existing *diagnoses*, treatments carried out so far, current test results, important family and social aspects, and the goals for medical rehabilitation. The report should state that physical and *mental* performance are threatened in the long term, and that social participation appropriate to the child's developmental stage is at risk. The report may be supplemented by additional diagnostic documentation. Doctors can use form G0600 to invoice their fee for completing the report.

2. APPLYING TO THE COST-COVERING AGENCY (KOSTENTRÄGER)

The second step is to fill in form G0200, the application for benefits relating to medical rehabilitation for children and adolescents (Antrag auf Leistungen zur Rehabilitation für Kinder und Jugendliche (Kinderrehabilitation). You must then send all three completed forms (G0200, G0600 and G0612) to German Pension Insurance

(Deutsche Rentenversicherung). It takes approximately four weeks to review your application. Clearly structured information about the application can be found in German at
www.deutsche-rentenversicherung.de/DRV/DE/Reha/Medizinische-Reha/Reha-fuer-Kinder-und-Jugendliche/reha-fuer-kinder-und-jugendliche.html.

PRACTICAL TIPS FOR COMPLETING THE APPLICATION FORM

Application G0200 consists of a total of six pages, which must be completed in full. The insurance number (Versicherungsnummer) of the person whose statutory pension fund membership the application for rehabilitation is based on (usually an employed parent) must be entered at the top left of each page. If you have downloaded the forms from the German Pension Insurance (Deutsche Rentenversicherung) website, the PDF document's fields can be filled in on the computer. You can save the document on your own computer. After printing, the application must be signed by hand by the applicant (a parent or their legal representative, or a legal guardian). Of course, it is also possible to fill in all fields by hand. Please ensure, however, that the handwriting is easily legible.

In the application form, information belonging to the same topic is organised in numbered blocks. The application form comprises the following blocks:

1. **Personal details of the person whose statutory pension fund membership the application for the rehabilitation benefit is based on (e.g. a parent):** this must be the same person whose insurance number is entered at the top left of the form.

2. **Personal details of the child:** the child who is to receive medical rehabilitation for children and adolescents.

3. **Accompanying person:** mark the appropriate box to indicate whether someone will accompany the child. If a child older than 12 years is to be accompanied, this must be substantiated as medically indicated. Any supporting documents should be attached to the application. The doctor who is writing the *medical report* will provide this evidence.

4. **The child's health insurance provider:** form of health insurance (statutory or private) and details (e.g. address)

5. **Child's treating doctor**: the doctor who is filling in the *medical report.*

6. **Further details regarding the child:**

6.1 If the child has lost one or both parents and receives a state orphan's pension (Waisenrente), or if this type of social benefit has been applied for, mark this box and enter the insurance number.

6.2 If the child is already employed in a way that entitles it to German civil service benefits, mark 'yes' and enter the employer's name and address. Please note: civil servants with state benefit entitlements are unable to receive rehabilitation benefits through German Pension Insurance (DRV). Untenured, trainee civil servants (Beamt*innen auf Widerruf) and those within the probationary period (Beamt*innen auf Probe), as well as public service employees with supplementary benefit entitlements (Zusatzversorgung) are not affected by this exclusion.

6.3 Possible reasons for the *rehabilitation need* are listed here. If one of them applies, mark 'yes'. In this case, you must also enter the name of the authority that officially recognised this reason, including the associated file number. In such cases, the application may be forwarded to another cost-covering agency, e.g. statutory accident insurance (Unfallversicherung). This does not affect your child's entitlement to medical rehabilitation.

6.4 Information must be entered here if the *rehabilitation need* arose from an accident or through another person. If 'yes', fill in questionnaire F0870 as referred to in the form. You must also specify if a claim for damages has been filed. In such cases, the agency covering medical rehabilitation (Rehabilitationsträger) can make a claim against third parties. Again, this does not affect your child's entitlement to medical rehabilitation.

7. **The following details are only required if your child is at least 18 years old:** they concern school, vocational training (apprenticeship or traineeship), study, voluntary service and disabilities.

8. **Personal details of the person whose statutory pension fund membership is the basis for the application:** details regarding insurance status, pension contributions paid abroad, and employment details.

9. **Application by another person on behalf of the child:** if parents are unable or lack the cognitive ability to make an application, a legal representative, a legal guardian or a person whom the parents have granted power of attorney can also take on this task. In these cases, the power of attorney or court order must also be submitted.

10. **Communication devices and approved medical aids:** all devices a child needs to manage everyday life, and therefore also needs during rehabilitation, should be listed here. These include, for example, glasses, hearing aids and prostheses.

11. **Document access:** this is an option for transferring documents electronically, using the German government's 'De-Mail' system (secure email access – must be applied for with an approved provider). People with a disability can also mark a box to request documents in an additional format (e.g. in Braille or as audio files).

12. **Explanation and information:** by signing the application, the applicants agree to data collection. They also release doctor's practices, health services and social service providers (e.g. statutory health insurance funds) from their professional confidentiality obligation. This way, the statutory pension fund (Rentenversicherung) can request further documentation from the providers mentioned in the *medical report (Befundbericht),* and then also make them available to experts involved in the assessment of the application. This may be crucial for the approval of the application. It also means that medical documents can be sent to the rehabilitation facility.

13. **Signatures:** these confirm that the application form was completed truthfully.

COMMON MISTAKES WHEN COMPLETING THE APPLICATION FORM

Providing incorrect or inaccurate information in the application process can lead to rehabilitation being denied. It is important to read the application form carefully in order not to miss anything.

Here is a list of the most common pitfalls:

- **Missing/incorrect insurance number:** this number can be found on your pay slip (it may be called 'Sozialversicherungsnummer' or 'Rentenversicherungsnummer', often abbreviated to 'SV-Nummer' and 'RV-Nummer' respectively).
- **Illegible entries if the form has been filled in by hand:** if the personnel at the processing agency or experts providing assessments are unable to read the information entered, details that are crucial for an application to be approved may be lost. Clear handwriting is therefore very important.
- **Missing contact details (telephone number, email address):** in principle, providing your contact details is voluntary. But please consider that not providing them makes it difficult to contact you for clarification. Answering clarifying queries may be an important step towards approval.
- **Missing details after marking a 'yes' box (e.g. at numbers 6, 8 and 11):** if questions are answered 'yes', additional details are often required, and providing them should not be overlooked.
- **Missing signatures:** all signature fields must be signed, or the application cannot be processed.
- **Revoking consent, e.g. revoking the release from medical confidentiality:** in principle, any form of consent may be revoked. If this, however, means a loss of information that is considered important to the approval of the application, it may lead to the application being rejected.
- **Missing documents:** all required documents should be submitted together.

If you have problems or questions regarding the application, health insurance providers and German Pension Insurance (DRV) service points can help you. You can find the service point closest to you at www.deutsche-rentenversicherung.de/DRV/DE/Beratung-und-Kontakt/Beratung-suchen-und-buchen/beratung-suchen-und-buchen_node.html.

3. REGISTERING WITH THE FACILITY:

If your application is approved, your statutory pension fund (Rentenversicherung) will send you an official notification letter, as well as information about the facility where rehabilitation will take place, and about the duration of your stay. It is best if you then call the facility to confirm the rehabilitation start date. This is also a good opportunity to discuss further questions regarding accommodation, school lessons and, if necessary, the accompanying person. Furthermore, parents have the opportunity to clarify personal requirements such as dietary habits in advance.

DEALING WITH AN APPLICATION BEING REJECTED

In rare cases, an application may be rejected initially. It is important to carefully read the reasons given. The following are common reasons for the rejection of an application:

- *Outpatient* options have not yet been exhausted, and acute treatment (e.g. in a hospital) is considered more appropriate.
- The person's health status is not expected to improve.
- The previous rehabilitation was fairly recent, and another intervention is not yet required.
- The requirements related to insurance law have not been met (see Chapter 4).

You should always lodge an *appeal* (Widerspruch) against the decision, and do so in writing. The time limit for lodging an *appeal* is normally four weeks, starting with the day you received the notice of rejection of your application (Ablehnungsbescheid). Make sure you check the time limit indicated in the legal notice (Rechtsmittelbelehrung). You can include your reasons with the *appeal* straight away, or lodge it without giving reasons in the first instance, e.g. in order to meet the deadline. In this case, you must resubmit the *appeal* including your reasons, also in writing, as soon as possible. On request, the cost-covering agency that rejected the application must grant access to their files. This is useful for obtaining additional information that may not be contained in the letter. A conversation with your treating doctor can help you find the right

wording for the *appeal* and argue against the reasons for the rejection. You should attach another medical certificate. If the *appeal* is successful, you receive an official *reversal notice* (Abhilfebescheid) and your child will be able to start medical rehabilitation.

In most cases, lodging an *appeal* is worthwhile. Support is available for wording the *appeal*, e.g. at German Pension Insurance (Deutsche Rentenversicherung) service points, or from independent social service providers, e.g. from the German Social Association (Sozialverband Deutschland). Please note that membership is required to receive services from the German Social Association. The argumentation should describe why the child's later *earning capacity (Erwerbsfähigkeit)* is at risk. This is because one of the important aims of medical rehabilitation through German Pension Insurance (Rentenversicherung) is to preserve *earning capacity*. If the *appeal* is also rejected, it is possible to consider filing a complaint with the Social Law Court (Sozialgericht).

Preferred facility

Patients have a 'right to preference and choice' (Wunsch- und Wahlrecht), whereby they can indicate which rehabilitation facility they would like to attend. Rehabilitation facilities sometimes differ significantly regarding the services they offer. In principle, German Pension Insurance (Deutsche Rentenversicherung) can only approve places in facilities with which it has a contract. In addition, the facility must be able to treat the presenting health conditions. If you have a particular preference, you should provide your reasons in an informal letter and include it with your application. If the application has been approved, but not your preferred facility, you can also *appeal* against that decision.

You can also find further information and tips for the application process in the downloads section at
www.mimi-reha-kids.de

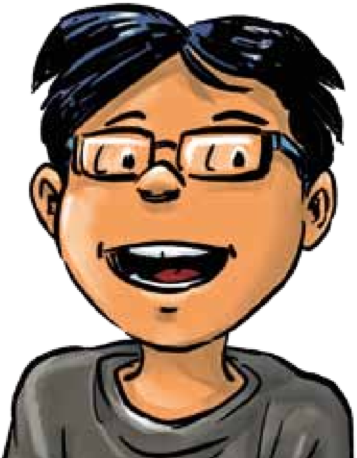

Case studies

To show what may happen during medical rehabilitation, a few fictional case studies are presented here:

CASE STUDY A: TARIK HAS ADHD

Tarik is ten years old. He lives in Berlin with his parents and his 16-year-old brother Ahmet. Tarik is often hyper and restless. If something doesn't work out, he can be quick to get angry. He also finds it very difficult to fall asleep at night because his thought processes just don't settle down.

Because of his behaviour, Tarik and Ahmet keep getting into fights with each other. Ahmet feels that everything is always just about Tarik. Some days, when the family is sitting down together, it's almost impossible for Ahmet to tell his parents about anything, because Tarik keeps interrupting and draws all the attention to himself with his constant fidgeting. Tarik's behaviour has already had consequences at school as well. He's been excluded from class several times because he was disrupting the lesson with his behaviour, and was starting arguments with other children.

On advice from the school, Tarik has been seeing a child and adolescent psychiatrist once a week for about the last two years. But because Tarik's behaviour continues to severely stress him and the other family members, the child and adolescent psychiatrist recommends medical rehabilitation. He also suggests that one of the parents should accompany Tarik in order to learn about how Tarik can be supported in everyday life.

Tarik's parents are therefore lodging an application for medical rehabilitation with German Pension Insurance (Deutsche Rentenversicherung). After four weeks, they receive a letter saying that their application has been rejected. Together with the child and adolescent psychiatrist, they discuss the rejection and, within the four-week time limit, they put together an *appeal*, in which they argue why the locally available *outpatient* services are not sufficient and that rehabilitation is medically indicated. After six weeks, the *appeal* is accepted and medical rehabilitation for Tarik approved. A few weeks later, he travels to the rehabilitation facility with his mother to stay for five weeks.

At the facility, Tarik learns to solve difficult and frustrating situations without coming into conflict with others, and to control his anger. New activities help him use his energy for the things he enjoys. After some time, Tarik is already able to better concentrate during lessons at the facility. Tarik's mother is also learning a lot of new information about dealing with Tarik's illness, and is better able to respond to his needs. She also more often succeeds in calming Tarik down before a situation escalates.

After staying at the rehabilitation facility for five weeks, Tarik and his mother return home. Tarik's mum has also been telling his dad and his brother Ahmet about everything they learned during rehabilitation. Now they all know how they can support Tarik. Since attending rehabilitation, Tarik has been calmer at home and doesn't get upset so quickly anymore. Even the fights with his brother and the problems he used to have at school have become markedly less frequent.

CASE STUDY B: ZAHRA HAS OBESITY

Fourteen-year-old Zahra lives in Hamburg with her mother. Even as a small child, Zahra already had a solid build, but by now she has become severely overweight. Zahra actually enjoys moving her body, but now she has pain in her knees more and more often. Zahra doesn't sleep well at night because she keeps thinking of the taunts she gets from the other children. Because of her lack of sleep, she lacks concentration during the day, and finds it hard to do her schoolwork.

She's generally no longer happy at school because her classmates make fun of her weight. More and more often she tells her mother in the morning that she isn't feeling well and therefore can't go to school. Instead, Zahra hunkers down in her room and plays on her smartphone or watches TV series.

The paediatrician has talked to Zahra and her mother several times about her weight problems. She is worried that her severe overweight will lead to other *chronic* conditions such as diabetes and high blood pressure. Because Zahra is also complaining about pain, and is withdrawing more and more often, the paediatrician and Zahra's mother lodge an application for medical rehabilitation with German Pension Insurance (Deutsche Rentenversicherung).

Two months after lodging the application, Zahra's medical rehabilitation begins at a facility on the Baltic Sea coast. Zahra stays at the facility for almost five weeks. During this time, she manages to reduce her weight. She gets to know a range of sports and movement exercises that she enjoys, and that she can do without being in pain. At the rehabilitation facility, Zahra has a lot of contact with children and adolescents who have similar experiences, and whom she can talk to about her fears and worries. At the facility, Zahra also learns how to prepare healthy and tasty meals.

On the morning of her last day, Zahra's mum arrives at the rehabilitation facility. Before they both drive back to Hamburg in the evening, Zahra's mother also talks once more with the doctors and therapists at the clinic. She finds out what to look out for regarding Zahra's diet, and how she can support Zahra with a healthy lifestyle.

Her rehabilitation stay has been good for Zahra. Her weight has dropped and she is exercising more. Sometimes she is still in pain, but she now knows which exercises she can still use to improve her health. Zahra and her mother are now also watching more closely what Zahra eats. Staying at the rehabilitation facility has made Zahra more confident, which is also helping her deal with the taunts from other young people. She is now going to school regularly, and also meets up with her girlfriends more often. She continues to catch up on the phone and by text message with her new girlfriends from the rehabilitation facility.

CASE STUDY C: JAKUB HAS ASTHMA

Jakub and his family live near Berlin. Jakub is 12 years old, his sister Hanna is 14 and his brother Piotr is 10. Jakub and his siblings ride skateboards and therefore like spending time outdoors. Because of his shortness of breath, however, Jakub can only rarely join his siblings. His nightly coughing fits also disturb his sleep, which means that Jakub is often tired during the day, and finds it hard to follow his lessons. So that he doesn't fall behind in his class, Jakub receives tutoring twice a week.

This situation isn't easy for Jakub. Some days he is too exhausted to join activities with his siblings or friends, and sometimes he doesn't have the time because he has to catch up on schoolwork. Jakub is feeling more and more isolated and less confident in his abilities.

While Jakub has been seeing a paediatric pneumologist for several years already, he is still not feeling better. The paediatric pneumologist and Jakub's parents have also noticed that he has been less and less confident lately, and has sought less contact with other children. They decide to lodge an application for medical rehabilitation for Jakub. It is intended to relieve Jakub's asthma symptoms and to strengthen his self-esteem.

After 3 weeks, Jakub's parents receive written approval for medical rehabilitation from their statutory pension fund (Rentenversicherung). In it, the pension fund is recommending a facility on the North Sea coast. After checking back with their paediatric pneumologist, Jakub's parents accept the recommendation.

Four weeks later, they're off. The train trip to the facility, where he will be staying accompanied by his father, is paid for by German Pension Insurance (Deutsche Rentenversicherung).

At the rehabilitation facility, Jakub's medication is adjusted in consultation with his paediatric pneumologist. He is also receiving medication that he can take before engaging in strenuous physical activity, to protect him from coughing fits and shortness of breath as a result of the exertion. During his stay at the facility, Jakub is also talking to a psychologist about his fears and worries. During his respiratory *physiotherapy* sessions, he is learning new breathing techniques that help him improve his breathing and body awareness. At the end of the rehabilita-

tion, Jakub's health is significantly better because of all the different interventions. It also managed to increase his self-esteem and his confidence in his own body.

Back home, Jakub is out and about on his skateboard again more often with his siblings. He is sleeping better at night and has fewer problems at school. Instead, he is more often spending time with other children in his class.

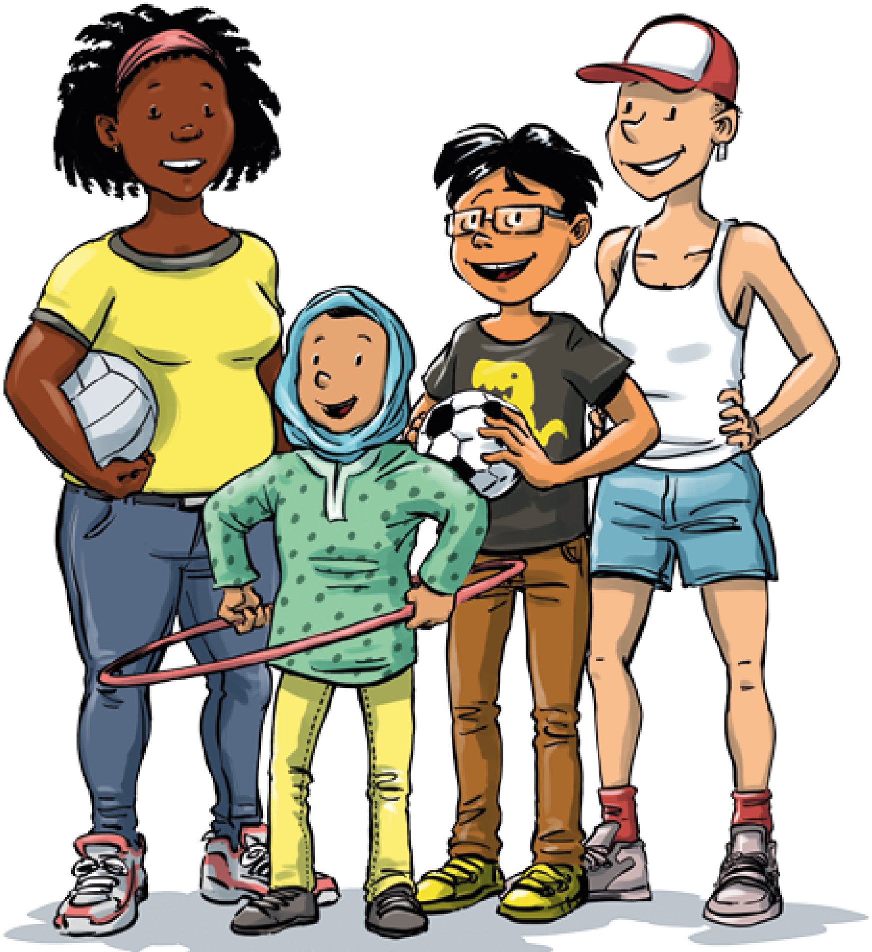

Frequently asked questions (FAQ)

When are children and adolescents entitled to medical rehabilitation?
Children and adolescents are entitled to medical rehabilitation paid for by German Pension Insurance (DRV) if the following criteria are met:

- A *positive rehabilitation prognosis:* there must be a good chance that medical rehabilitation can help your child.
- *Rehabilitation need:* your child must be suffering from a *chronic* illness that is limiting or endangering the child's health. If the *chronic* condition is clearly limiting your child's social life, and may be putting its later *earning capacity* at risk, *rehabilitation need* is established.
- *Rehabilitation capacity:* your child must be physically, *psychologically* and socially capable of participating in medical rehabilitation.

For the costs to be covered by German Pension Insurance (DRV), certain criteria relating to insurance law must also be met. However, you can always lodge an application with German Pension Insurance (DRV) in the first instance. If German Pension Insurance (DRV) is not the responsible agency in your particular case, the staff will forward the application to another agency that can also cover medical rehabilitation, which will then process the application.

Who do I contact?
Your first port of call is the doctor who is treating your child. However, other support services are also available. At the end of this guide, you will find a services directory with additional contact information.

Who is considered a 'child'?
Apart from biological children, this also includes stepchildren, foster children, and grandchildren who have become part of the household.

Is the application form available in languages other than German?
No. The German Pension Insurance (DRV) application form is available in German only.

What do I do if my application is rejected?
On the one hand, you can *appeal* the decision (See Chapter 5). On the other hand, you can – if rehabilitation is not medically indicated at the time – find out about prevention and health promotion services available near you (e.g. through your health insurance provider) and take advantage of those.

Can I choose the facility where my rehabilitation will take place?
You have a right to 'preference and choice' (Wunsch- und Wahlrecht). This means that you can indicate a preference regarding the rehabilitation facility as part of the application. The statutory pension

fund assesses the information and the reasons submitted by the insured person, and takes them into account where possible.

Do I have to take care of travel to and from the facility myself?
Yes. German Pension Insurance covers the costs of 2nd class rail travel and public transport. If you arrive by car, up to 20 cents per kilometre driven on the way there and back are reimbursed, with the total amount capped at 130.00 euros for *inpatient* rehabilitation.

Will an interpreter be provided?
An interpreter will be provided on request for the initial medical consultation at the rehabilitation facility, where you will, for example, discuss your child's treatment plan with the doctors, but not for subsequent treatment interventions.

Up to what age can a person access medical rehabilitation for children and adolescents?
In certain cases, young adults up to the age of 27 years can apply for medical rehabilitation for children and adolescents. This applies if they are in vocational training (apprenticeship or traineeship), or serving as part of the government's 'social or ecological year' programme (Freiwilliges Soziales oder Ökologisches Jahr) or the Federal Voluntary Service (Bundesfreiwilligendienst). People with a disability who are unable to support themselves independently can also access medical rehabilitation for children and adolescents up to the age of 27 years.

Does it cost money to attend medical rehabilitation for children and adolescents?
No, you will not incur any costs if your child attends medical rehabilitation covered by German Pension Insurance (DRV).

Will my child receive school lessons while staying at the facility?
Yes, lessons will take place at the facility in order to ensure that your child won't fall behind while staying there.

Can I accompany my child to the rehabilitation facility?
Children up to the age of 12 years may be accompanied by one other person (one of the parents in most cases). If medically indicated, older children may also be accompanied.
The aim of rehabilitation with an accompanying adult is to give your family more strength to handle the challenges of everyday life in the long term – through attending treatment interventions, training courses and exercise classes together. The focus, however, is always on the young patient. This means that accompanying adults do not receive treatment for their own personal problems. If required, however, parents can talk to individual therapists about questions they may have on particular aspects of bringing up children with a chronic illness and their siblings.
Joint activities for all accompanying adults (e.g. discussion groups and leisure activities) are also offered. As it is for your child, it may also be helpful for you to make contact with parents in similar situations.

As an accompanying person, will my salary continue to be paid for the duration of rehabilitation?
For the time you are staying at the facility, you can't go to work or look after your household and other children you may have. So that you can still accompany your child, your statutory pension fund (Rentenversicherung) will, under certain conditions, reimburse you for lost income. Livelihood support benefits such as unemployment benefit II (ALG II) also continue to be paid. The cost of hiring a person to provide you with household assistance may also be reimbursed. You can apply for these benefits with your statutory pension fund (Rentenversicherung) in advance.

Who looks after the rest of my family while I am at the rehabilitation facility as an accompanying person for my child?
If required, household assistance and/or child care will be covered for your family by German Pension Insurance (DRV), as long as certain criteria are met. Alternatively, siblings may also be brought along to the facility.

Is it possible to pray at the rehabilitation facility?
Yes. Many rehabilitation facilities have prayer rooms and quiet rooms. You can also pray in your own room at any time.

What if my child has particular dietary habits (vegan/vegetarian/halal etc.)?
It is best to clarify such questions with the facility by telephone before the start of rehabilitation. The staff will consider your requests as far as possible.

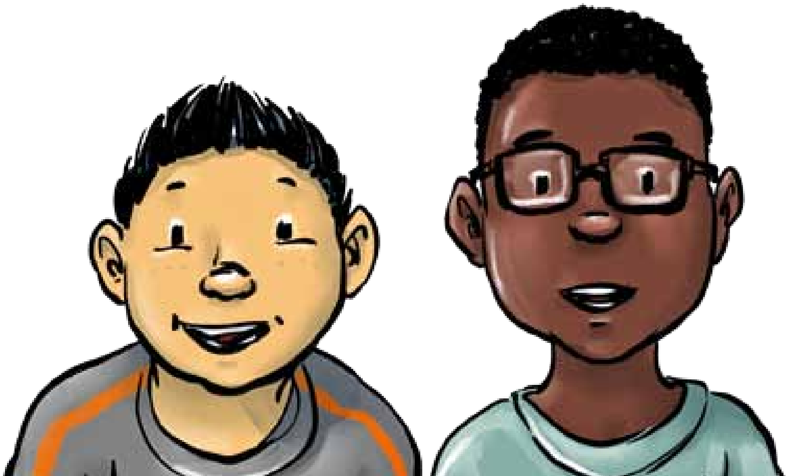

Services directory

Federal and regional agencies of German Pension Insurance (Deutsche Rentenversicherung)

Deutsche Rentenversicherung Bund (Federal German Pension Insurance Fund)
Ruhrstraße 2 · 10709 Berlin
Ph.: 030 8650
Fax: 030 86527240
Email: drv@drv-bund.de

Deutsche Rentenversicherung Knappschaft-Bahn-See (German Pension Insurance, Mines, Rail and Shipping Fund)
Pieperstraße 14 – 28 · 44789 Bochum
Ph.: 0234 304 0
Fax: 0234 30466050
Email: zentrale@kbs.de

Deutsche Rentenversicherung Berlin-Brandenburg (German Pension Insurance, Berlin-Brandenburg Fund)
Bertha-von-Suttner-Straße 1
15236 Frankfurt (Oder)
Ph.: 0335 551 0
Fax: 0335 551 1295
Email: post@drv-berlin-brandenburg.de

Deutsche Rentenversicherung Nord (German Pension Insurance, Northern Regional Fund, main office)
Ziegelstraße 150 · 23556 Lübeck
Ph.: 0451 485-0
Fax: 0451 485 15333
Email: info@drv-nord.de

Deutsche Rentenversicherung Nord (German Pension Insurance, Northern Regional Fund, Hamburg Branch)
Friedrich-Ebert-Damm 245
22159 Hamburg
Ph.: 040 5300-0
Fax: 040 5300-14999
Email: info@drv-nord.de

Kinder-Reha-Hotline der DRV Nord (children's rehabilitation helpline of German Pension Insurance, Northern Regional Fund):
Since 2017, qualified counsellors at German Pension Insurance's Northern Regional Fund (DRV Nord) have been responding to families' and doctors' questions regarding medical rehabilitation for children and adolescents from Monday to Friday between 9 am and 12 noon on 0451 485-25999.
Outside these operating hours, callers can leave a message including name and telephone number on the answering machine. The counsellor at DRV will call back on the next working day at the latest.

Service points (Auskunft- und Beratungsstellen) of Deutsche Rentenversicherung (German Pension Insurance)

www.deutsche-rentenversicherung.de/DRV/DE/Beratung-und-Kontakt/beratung-und-kontakt_node.html
Search option by postcode or city/town available.

Information sources regarding medical rehabilitation for children and adolescents

www.kinder-und-jugendreha-im-netz.de
The Bündnis Kinder- und Jugendreha e.V. (BKJR) (Child and Adolescent Rehabilitation Alliance) offers clearly structured, very comprehensive information on medical rehabilitation for children and adolescents. The website also includes an overview of rehabilitation facilities in Germany that specialise in child and adolescent rehabilitation.

Website URLs

www.mimi-reha-kids.de
Further information and tips on the topic of rehabilitation for children and adolescents are available on the project website of the Mimi-Reha-Kids project.

www.deutsche-rentenversicherung.de
Information on pensions, medical and occupational rehabilitation is available on the webpages of Deutsche Rentenversicherung (German Pension Insurance). The counselling centres of Deutsche Rentenversicherung (German Pension Insurance) can also be found there.

www.bmas.de/DE/Themen/Teilhabe-Inklusion/Rehabilitation-und-Teilhabe/rehabilitation-und-teilhabe.html
Rehabilitation and participation for people with disabilities are a focus area of the Bundesministerium für Arbeit und Soziales (BMAS) (Federal Ministry of Labour and Social Affairs).

www.bmg.bund.de
The website of the Bundesgesundheitsministerium (Federal Ministry of Health) contains information on social insurance providers, and on the services and benefits they offer.

Looking for a suitable rehabilitation facility

On its website, the **BKJR (Child and Adolescent Rehabilitation Alliance)** provides a list of rehabilitation facilities specialising in child and adolescent rehabilitation.
www.kinder-und-jugendreha-im-netz.de/reha-kliniken-fuer-kinder-jugendliche/
The treatment specialisations of individual facilities can be found at www.kinder-und-jugendreha-im-netz.de/fileadmin/pdf/KJR_Klinikliste/Kinder-und-Jugendreha-im-Netz_Klinikliste.pdf.

Outpatient rehabilitation facilities for children and adolescents

REHA VITA in Cottbus
Outpatient rehabilitation facility for children and adolescents with a focus on obesity and psychiatric disorders
www.reha-vita-online.de/reha-vita/ambulante-reha/ambulante-reha-fuer-kinder-und-jugendliche

UniReha – Zentrum für Prävention und Rehabilitation der Uniklinik Köln (Centre for Prevention and Rehabilitation at Cologne University Hospital)
Outpatient rehabilitation facility for children and adolescents with a focus on neurological and orthopaedic conditions, as well as on obesity
https://unireha.uk-koeln.de/kinder-jugend-reha/ambulante-rehabilitation-amlor/

Counselling centres

Germany-wide

Unabhängige Patientenberatung Deutschland (Independent Patient Advice Service Germany)
Germany-wide advice for patients on a wide range of health issues, including rehabilitation
www.patientenberatung.de/de/beratungsangebot

Berlin

ajb gmbh –Gemeinnützige Gesellschaft für Jugendberatung und psychosoziale Rehabilitation (Charitable Association for Youth Counselling and Psychosocial Rehabilitation)
This agency offers holistic counselling and therapeutic services, accommodation and employment, learning and social contact for people with special support needs, people in crisis situations, special life circumstances or with a mental illness.
www.ajb-berlin.de/Startseite.26.0.html

AWO Berlin Kreisverband Südost e.V. (Berlin Worker's Charitable Association, Southeast District Inc.)
Şifahane – health and migration counselling
Support and counselling on personal and health problems as well as integration into the health system.
www.awo-südost.de/sifahane

Berliner Zentrum für Selbstbestimmtes Leben e.V. (Berlin Centre for Independent Living Inc.) – specialist unit for refugees with disabilities and older refugees
Counselling and support for refugees with a disability or *chronic* illness. Context-oriented social work assistance, psychosocial counselling and support with claiming entitlements under social law, as well as assistance with applications.
www.bzsl.de/bns.html

Diakoniewerk Simeon gGmbH (Simeon Diaconal Charitable Works Inc.) – Neighbourhood Mothers in Neukölln
Trained non-German mothers inform other mothers on topics such as parenting, the education system and health, and provide referrals to concrete assistance and information for families.
www.diakoniewerk-simeon.de/beratung-integration/stadtteilmuetter-in-neukoelln

EUTB – Ergänzende unabhängige Teilhabeberatung (Supplementary Independent Participation Counselling)
Free support and advice for all people with disabilities and people at risk of disability, but also their relatives, on all issues related to participation.
www.teilhabeberatung.de

Die Insel e. V. (The Island Inc.)
Counselling, information and referrals for chronically ill children, adolescents, their families and supporting professionals
www.kompaxx.de/die-insel/kontakt

Integrationslotsen*innen (Integration Pilots)
Integration pilots support migrants in a range of languages across all districts of Berlin.
www.berlin.de/lb/intmig/themen/integrationslots-innen/traeger

InterAktiv e. V. (InterActive Inc.) in Berlin
offers people with a disability and a migration or refugee background and their relatives individual, culturally sensitive and family-oriented counselling services in Turkish, Arabic, Russian, English and French.
www.interaktiv-berlin.de/de/beratung

Interkulturelles Beratungs- und Begegnungs-Centrum IBBC e. V. (Intercultural Counselling and Drop-In Centre Inc.)
Support, counselling, care and assistance for children, adolescents and adults. Intercultural and prevention-oriented.
www.ibbc-berlin.de/index.html

Youth migration services in Berlin
Counselling on protecting livelihoods (ALG II, BAföG) and health, among other issues
www.caritas-berlin.de/beratungundhilfe/berlin/flucht-und-migration/beratung/fuer-junge-migranten-bis-27-jahre/jugendmigrationsdienst

knw Kindernetzwerk e. V. (Children's Network Inc.)
Counselling and support services for families with a chronically ill or disabled child or young adult.
www.kindernetzwerk.de/de

Medizin Hilft e. V. (Medicine Assists Inc.) (open.med Berlin)
Medical care for people with no or restricted access to the public health system. Referral to general or specialist medical care.
www.medizin-hilft.org/de

MINA – Leben in Vielfalt e. V. (Life in Diversity Inc.) in Berlin
Counselling and self-help groups for parents of children with disabilities in Arabic and Turkish.
www.mina-berlin.eu

Notdienst für Suchtmittelgefährdete und -abhängige Berlin e. V. (Berlin Emergency Service for People at Risk of or Dependent on Addictive Substances Inc.)
Counselling centre for people with addictions, including interpreting service for Arabic, Farsi and Turkish.
www.drogennotdienst.de

Polnischer Sozialrat e. V. (Polish Social Council Inc.) in Berlin
Counselling in Polish on the topic of rehabilitation
www.polskarada.de/porady/gesundheit/?lang=de

Sozialpädiatrisches Zentrum der Charité (Social Paediatric Centre at Charité Hospital)
Treatment and assistance (training courses) for children and adolescents with a chronic illness and their families
www.spz.charite.de

TransVer – Psychosoziales Ressourcen-Netzwerk zur interkulturellen Öffnung (Psychosocial Resource Network for Intercultural Development)
Information and referral to psychosocial and other appropriate services for people with a migration and/or refugee background.
www.transver-berlin.de

Zentrum für Interkulturelle Psychiatrie & Psychotherapie (ZIPP) (Centre for Intercultural Psychiatry & Psychotherapy)
Treatment or referral to general psychiatric and psychotherapeutic services, as well as to general, or to migration and refugee-specific services of the psychosocial care system.
http://psychiatrie-psychotherapie.charite.de/fuer_patienten/ambulanzen/zentrum_fuer_interkulturelle_psychiatrie_psychotherapie_zipp

Hamburg

ABeSa – ambulante Hilfen GmbH (Outpatient Assistance Ltd.) is an agency of the Eingliederungshilfe (Integration Assistance) as well as the Jugend- und Familienhilfe (Youth and Family Assistance) in Hamburg.
Focus on support for people from the age of 14 years who have a mental health condition
Support and counselling taking into account diverse cultural backgrounds
www.abesa-hh.de/ueberuns.html

Amnesty for Women e.V. in Hamburg
Counselling in German, Spanish, Thai, English and Swahili on issues of social benefit entitlements (unemployment benefit I and II, social security benefit, child benefit, parents’ benefit and care benefit – Arbeitslosengeld I und II, Sozialhilfe, Kindergeld, Elterngeld, Pflegegeld)
www.amnestyforwomen.de/deutsch/beratung

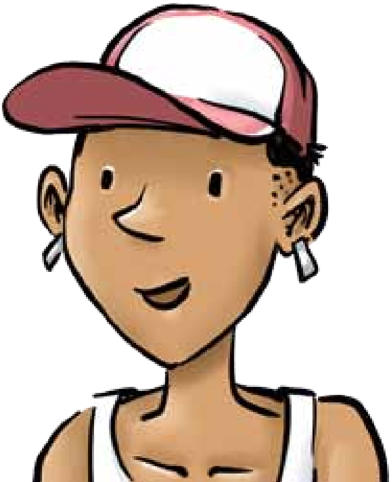

BBZ Bildungs- und Beratungszentrum (Education and Counselling Centre) in Hamburg
Counselling and support services for parents and pupils who are unable to attend school for extended periods because of illness.
https://bbz.hamburg.de/paedagogik-bei-krankheit/

Children for Tomorrow – Universitätsklinikum (University Hospital) Hamburg-Eppendorf
Outpatient psychiatric, psychotherapeutic and psychosocial care for refugee children in Hamburg
www.children-for-tomorrow.com

Flüchtlingszentrum (Refugee Centre) Hamburg – Clearingstelle (Clearinghouse)
Supporting foreigners with clarifying health insurance status and with integration into the general health and social services systems.
www.fluechtlingszentrum-hamburg.de/de/projekte/clearingstelle_mv.php

IN VIA Hamburg e. V. – language and cultural interpreters
Support through volunteer interpreters for appointments with public authorities, child care centres and schools, or for other advisory service or counselling appointments.
www.invia-hamburg.de/migration/sprach-und-kulturdolmetscher.html

JHJ Hamburg
Individual services and counselling in a range of languages such as English, Russian, Spanish, Turkish and Kurdish
www.jhj-hamburg.de/angebote

Youth migration services in Hamburg
Counselling and interpreting support in German, English, French and Spanish on issues related to school, vocational training, employment, dealing with public authorities, health etc.
www.invia-hamburg.de/migration/jugendmigrationsdienst.html

Polyklinik Veddel
Neighbourhood health centre with general practice, social and health counselling, psychological counselling and legal counselling services provided by the Refugee Law Clinic.
www.poliklinik1.org/konzeptvision

Psychosocial drop-in and counselling centres in Hamburg
Counselling and a range of services in the neighbourhood, e.g. native language services, addiction counselling, services on the topic of mental health and addiction, parents with a mental health condition and their children
www.psk-hamburg.de

Tumaini e. V.
Advice on support services and support with/accompanying service to public authority, school and medical appointments.
www.tumaini-ev.de

Notes

Glossary

**Addictive disorders (Suchterkrankungen)**	Disorders that are characterised by an uncontrollable craving for certain substances (such as alcohol or drugs) or activities (as in gambling, computer or shopping addiction).
**Appeal (Widerspruch)**	A legal means or recourse (Rechtsbehelf) against administrative decisions (i.e. taking action against a decision made by a public authority). If your application for rehabilitation is rejected, you will receive a notice of rejection (Ablehnungsbescheid). You can lodge an appeal (Widerspruch) against this rejection within a certain limited period (Frist).
**Chronic (chronisch)**	Long-lasting or developing slowly.
**Depression (Depression)**	A mental health disorder characterised by a low mood.
**Diagnosis (Diagnose)**	Identification of an illness
**Earning capacity (Erwerbsfähigkeit)**	The capacity to pursue a paid occupation. Earning capacity is reduced if the person can work only partially or not at all, due to a psychological or physical impairment.
**Induction to medical aids (Hilfsmitteltraining)**	Practising under instruction how to use a medical aid (such as a prosthesis), in preparation for using it in everyday life.
**Inpatient (stationär)**	Patients are accommodated in a health care facility such as a hospital or a rehabilitation facility. They stay at the facility overnight and also receive their meals there.
**Kur**	A sometimes still colloquially used, but now obsolete German term for rehabilitation.
**Medical report (ärztlicher Befundbericht)**	A document written by a medical doctor, containing a description of the patient's state of health.
**Mental**	see 'psychological'
**Occupational therapy (Ergotherapie)**	A type of therapy that promotes a person's everyday living skills.
**Outpatient, outpatient treatment (ambulante Therapie)**	Treatment where the patient does not stay overnight at the health care facility, but returns home on the same day.

Physiotherapy (Physiotherapie, Krankengymnastik)	A type of therapy that improves or preserves the functioning of the musculoskeletal system.
Psychological, psychiatric, mental (psychisch)	Concerning the state and/or health of the mind (psyche).
Psychosomatic (psychosomatisch)	This term is used when physical complaints have psychological causes.
Psychotherapy (Psychotherapie)	A type of therapy used to treat mental health disorders or the psychological consequences of physical illness.
Rehabilitation capacity (Reha-Fähigkeit)	A person must be physically and mentally capable of participating in rehabilitation; an important personal requirement for accessing rehabilitation benefits.
Rehabilitation need (Reha-Bedürftigkeit)	The person lodging the application must be in need of rehabilitation. This is a requirement for accessing rehabilitation benefits.
Rehabilitation prognosis (Reha-Prognose)	Prediction concerning the expected success of a rehabilitation intervention.
Reversal notice (Abhilfebescheid)	A letter issued by a public agency or authority that previously rejected an application, stating that the appeal (Widerspruch) against the rejection has been accepted. In case your application for rehabilitation has been rejected, you can lodge an appeal against the decision within a certain period. If your appeal is accepted, you receive a reversal notice (Abhilfebescheid). The reversal notice represents an administrative decision in favour of the insured person and approves the rehabilitation benefit.
Social insurance (Sozialversicherung)	All persons who are in types of employment that are subject to compulsory social insurance make contributions to the German statutory social insurance system. Certain risks (e.g. unemployment or illness) are borne collectively by all insured persons. The German statutory social insurance system consists of statutory health insurance, statutory accident insurance, statutory pension insurance, statutory unemployment insurance and statutory care insurance. These services are provided by a range of government-approved individual pension funds and insurance providers.

Acknowledgements

We thank the following experts who contributed significantly to the development of the Mimi-Reha-Kids project and the creation of this guide:
Dr. Stefan Berghem, Dr. Ulrich Eggens, Sidonie Fernau, Dr. Nathalie Glaser-Möller, Dr. Hatice Kadem, Vera Kleineke, Dr. Ingo Menrath, Dr. Betje Schwarz, Soner Tuna

The Ethno-Medical Centre Inc. (Ethnomedizinisches Zentrum e. V.) also provides other native language brochures on a range of health topics. They can be ordered or downloaded at www.mimi-bestellportal.de

- Health for all – a guide to the German health care system
- SARS-CoV-2 Coronavirus – Information and practical advice
- Health guide for asylum seekers in Germany
- Trauma induced disorders and Post-Traumatic Stress Disorder (PTSD)
- Depression (editions for Bavaria and Lower Saxony)
- Guide to computer, internet and gambling addiction (editions for Schleswig-Holstein and Lower Saxony)
- Addiction is an illness – a guide for interested individuals, those affected and their loved ones
- AIDS and HIV – Current Knowledge, Protection and Treatment
- Protective Vaccination – A Multilingual Guide
- Maternal Health — Information and contact points
- Diabetes: Inform – Prevent – Act
- Hospice and Palliative Care – Available services and making provisions for the future
- Violence protection for women in Germany
- Violence protection in Germany – A guide for male refugees and new arrivals

Medical rehabilitation through German Pension Insurance:

Support for children and adolescents with chronic illness

This guide offers important information on the topic of medical rehabilitation for children and adolescents. It answers the following questions:

- What is medical rehabilitation for children and adolescents?
- Which types of illness are typically treated at rehabilitation facilities?
- What happens during a stay at a rehabilitation facility?
- Can I accompany my child to rehabilitation?
- Do the children receive school lessons?
- Who covers the costs of rehabilitation?
- How and where can I lodge an application?
- Where can I get further support if I still have questions?

This guide can be downloaded at www.mimi-bestellportal.de and is available in the following languages: Arabic, Bulgarian, German, English, Farsi/Persian, Kurdish (Kurmancî), Polish, Russian, Serbian/Croatian/Bosnian, Turkish

Received from:

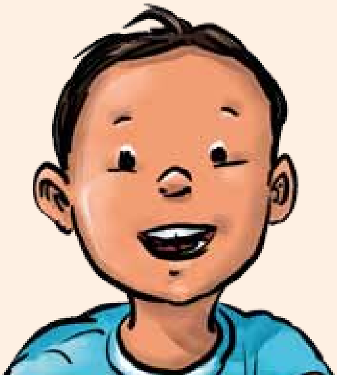

Supplement: Supplementary file 2 [file Data_Sheet_2.PDF]
