# Supplementary material for: Increasing the use of medical rehabilitation by children and adolescents with migrant background through a multimodal information campaign: protocol of a trend study and accompanying process evaluation (MiMi-Reha-Kids, DRKS00019090)
Source: Front Public Health. 2023 Jul 14;11:1089685. doi: 10.3389/fpubh.2023.1089685 (PMC10379645; doi:10.3389/fpubh.2023.1089685)
Supplement: Supplementary file 3 [file Data_Sheet_3.PDF]

Additional file 1: Items from the World Health Organization Trial Registration Data Set

**Data category**	**Information**
Register	German Clinical Trials Register
Last refreshed on	29 November 2021
Main ID	DRKS00019090
Date of registration	11/11/2019
Prospective Registration	Yes
Primary sponsor	Universität zu Lübeck, Institut für Sozialmedizin und Epidemiologie
Public title	Implementation and Evaluation of a Multilingual Information Campaign on Rehabilitation for Children and Young People from Migrant Backgrounds (MiMi-Reha-Kids)
Scientific title	Implementation and Evaluation of a Multilingual Information Campaign on Rehabilitation for Children and Young People from Migrant Backgrounds (MiMi-Reha-Kids)
Date of first enrolment	26/10/2020
Target sample size	1728
Recruitment status	Recruiting
URL	http://www.drks.de/DRKS00019090
Study type	Interventional
Allocation	Single arm study
Masking	Open (masking not used)
Control	Uncontrolled/Single arm
Assignment	Single (group)

Purpose	Health care system
Phase	N/A
Countries of recruitment	Germany
Contacts	Matthias Bethge<br>Ratzeburger Allee 160, 23562 Lübeck, Germany<br>+49 451 50051280<br>matthias.bethge@uksh.de<br>Universität zu Lübeck, Institut für Sozialmedizin und Epidemiologie
Key inclusion and exclusion criteria	Children and young people living in Berlin and Hamburg who participated in a medical rehabilitation program of the Federal German pension insurance and the regional German pension insurances Berlin-Brandenburg or North between 2019 and 2022 as well as their parents will be surveyed in the following year.
Health conditions or problems studied	Chronic health problems

Interventions	In the regions of Berlin and Hamburg, a multimodal information campaign will be conducted from the second quarter of 2020 to the end of the fourth quarter of 2022 to improve the use of child and youth rehabilitation by families with migration biographies. The campaign comprises the conception and dissemination of a guide to child and youth rehabilitation, the training of local mediators, information events for families from a migrant background, supplementary counselling hours and intercultural training for specialists in the field of rehabilitative care.
Primary outcome	The primary outcome of the trend study on utilization of rehabilitation services is the proportion of children and young people from migration backgrounds (basic set of indicators for mapping migrant status; Schenk et al. 2006). A migrant background is defined as a person who firstly migrated from another country and has at least one parent who was not born in Germany or secondly has two parents who migrated and/or do not have German nationality. It is expected that the proportion of children and young people with a migrant background will increase as a result of the information campaign.
Secondary outcomes	The following data are assessed as secondary outcomes or explanatory variables: Diagnosis from the rehabilitation discharge report, Health-related quality of life (KIDSCREEN-27; Ravens-Sieberer et al. 2005), Satisfaction with the rehabilitation, Sociodemographic data (for example gender, mother tongue, german language skills, living situation, social status)
Secondary ID(s)	19-299 (Ethics Committee University of Lübeck)<br>U1111-1241-4028 (Universal Trial Number)
Source of monetary support	Deutsche Rentenversicherung Nord (German Pension Insurance North), Deutsche Rentenversicherung Berlin-Brandenburg (German Pension Insurance Berlin-Brandenburg)
Status of ethics review	Approved
Approval date of ethics review	13/09/2019
