# Supplementary material for: Increasing the use of medical rehabilitation by children and adolescents with migrant background through a multimodal information campaign: protocol of a trend study and accompanying process evaluation (MiMi-Reha-Kids, DRKS00019090)
Source: Front Public Health. 2023 Jul 14;11:1089685. doi: 10.3389/fpubh.2023.1089685 (PMC10379645; doi:10.3389/fpubh.2023.1089685)
Supplement: Supplementary file 4 [file Data_Sheet_4.PDF]

Deutsche Rentenversicherung Berlin-Brandenburg

Deutsche Rentenversicherung Nord

Deutsche Rentenversicherung Bund

Study information

Research project "Implementation and evaluation of a multilingual information service on rehabilitation for children and adolescents with a migration background (MiMi-Reha-Kids)"

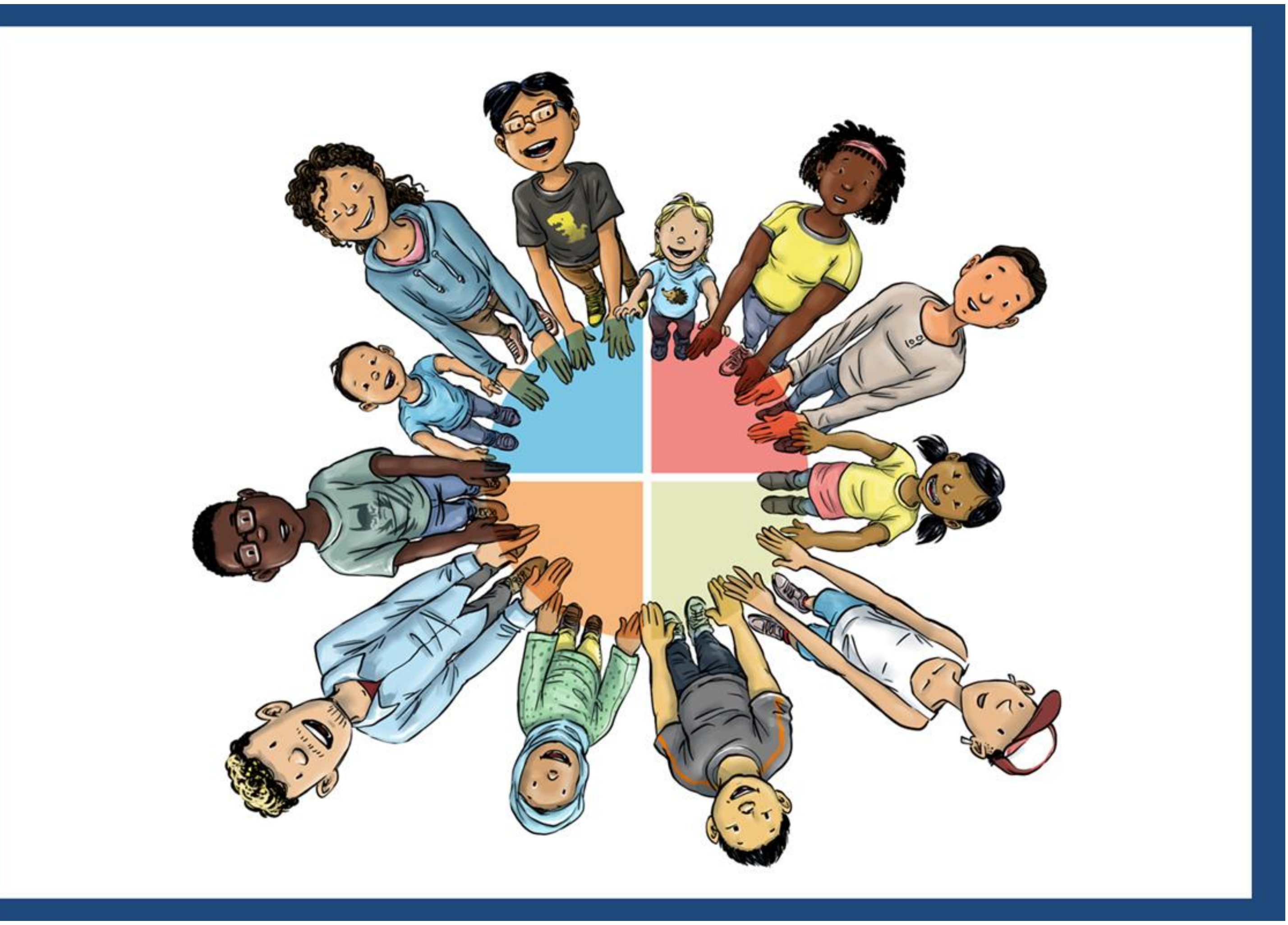

Ethno-Medizinisches Zentrum e.V.

Universität zu Lübeck
Institut für Sozialmedizin und Epidemiologie

Dear Parents,

We would like to inform you and your child about the "Implementation and evaluation of a multilingual information offer for rehabilitation for children and adolescents with migration background (MiMi-Reha-Kids)" study, and ask you to participate together with your child.

What is the study about?

You may wonder why we are sending this letter to you and your child in particular. Together with yourselves, more than 1000 families will receive the same letter this year. These are all families in Berlin and Hamburg with a child who received medical rehabilitation through German Pension Insurance last year. German Pension Insurance Berlin-Brandenburg and German Pension Insurance North, together with the Ethno-Medical Center, have started an information campaign to inform people about medical rehabilitation for children and young people. In this context, we, the researchers at the University of Lübeck, would like to learn a little more about the life situation of children and adolescents, and their parents, who have already made use of medical rehabilitation.

Further information under: **www.mimi-reha-kids.de**

How does the study work?

You will receive two questionnaires and a consent form with this letter. The blue questionnaire (E) is for you as the parent, under whose insurance account your child's medical rehabilitation is filed. The orange questionnaire (K) is for your child, who has received rehabilitation in the past year. If your child is younger than 8 years old or unable to fill out the questionnaire, it is sufficient if you only fill out the parent questionnaire.

What to do?

Participation in the study is voluntary. Neither participation nor non-participation will result in any disadvantages for you or your child. You can withdraw your participation at any time without giving reasons. If you choose to participate, we ask you to do the following. Ask your child to fill out the orange questionnaire (K). You, as the parent and insured, please complete the attached blue questionnaire (E) and consent form. Your consent form allows us to merge the questionnaire data with data from your insurance account. Place the consent form in the small envelope (T). Please send the completed questionnaires in the enclosed large return envelope (F) to the University of Lübeck. Please send the consent form in the small return envelope (T) to German Pension Insurance. You can simply drop the return envelopes in a mailbox, there is no cost to you. If you decide not to participate in the study, you do not need to send anything.

What data is collected?

Data on sociodemographics, attitudes and behaviors, and quality of life will be collected from the questionnaire pseudonymously. With your consent, the questionnaire data will subsequently be merged with the pseudonymized routine data (e.g., age of the child, gender of the child, approval diagnosis, period of your child's rehabilitation) from your insurance account.

How is the data collected?

The responsible parties work in accordance with the provisions of the General Data Protection Regulation (DS-GVO). German Pension Insurances Berlin-Brandenburg, North and Federation create a study number for insured persons. The study number is stored together with the insurance number in a protected study list at the pension insurance institution. The researchers cannot access the study list. The study number is entered on the questionnaires. The researchers register the questionnaires received on the basis of the study numbers. If the questionnaires have not been received after 4 weeks, the responsible pension insurance institution sends one reminder letter. To help them send the reminder letter, the researchers send a list with the questionnaires received so far to the pension insurance institution. Not completing the questionnaire, or not doing so until after receipt of the reminder letter, will not result in any disadvantages with regard to future benefits. No information will be transmitted from the pension insurance institutions to the researchers without the insured's consent. A record of routine data will only be generated by the pension insurance provider and linked to the study number for people who have given their written consent. A copy of this dataset is then created and cleansed of personal data (e.g., insurance numbers). This pseudonymized version is transmitted to the researchers at the University of Lübeck in an encrypted form, and merged with the pseudonymized data from the questionnaires on password-protected computers at the Institute of Social Medicine and Epidemiology at the University of Lübeck. After merging the data, the study number is replaced by a random sort number and the data is thus anonymized. Interests worthy of protection in the sense of § 75 paragraph 1 SGB X are not affected.

What happens after the evaluation?

All study data will be completely deleted after 10 years at the latest. The publication of research results in journals or books, or at conferences takes place exclusively in an anonymized form, and at no time allows conclusions to be drawn about you or your child as recognizable persons.

Who is responsible?

Prof. Dr. Matthias Bethge from the University of Lübeck is responsible for the project and data processing.

What are your rights?

Consent to the processing of your data is voluntary. You can revoke this consent at any time without giving reasons. The legality of the processing of the personal data carried out on the basis of the consent until the revocation is not affected by the withdrawal. You also have the right to receive a free copy of information about the data concerning you. you may also request the correction or deletion of your data. Your data can only be corrected or deleted before it is anonymized.
In the event of a complaint, please contact:

Unabhängiges Landeszentrum für Datenschutz Schleswig-Holstein
Holstenstraße 98, 24103 Kiel
E-Mail: mail@datenschutzzentrum.de

Request participation

Research thrives on the participation of people whose experiences form the cornerstone for changes and further developments in health care. The success of the study depends on a high level of participation by children, adolescents and parents. The more families participate, the more meaningful the results will be. We therefore ask you to decide whether you would like to participate.

If you have any questions, please contact Hannes Banaschak at the University of Lübeck.

We hope you will choose to participate, and thank you very much in advance for your support.

Hannes Banaschak
Wissenschaftlicher Angestellter
Universität zu Lübeck
Ratzeburger Allee 160
23562 Lübeck
Telefon 0451 500-51286
hannes.banaschak@uksh.de

Prof. Dr. Matthias Bethge
Studienleitung
Universität zu Lübeck
Ratzeburger Allee 160
23562 Lübeck
