# Supplementary material for: Increasing the use of medical rehabilitation by children and adolescents with migrant background through a multimodal information campaign: protocol of a trend study and accompanying process evaluation (MiMi-Reha-Kids, DRKS00019090)
Source: Front Public Health. 2023 Jul 14;11:1089685. doi: 10.3389/fpubh.2023.1089685 (PMC10379645; doi:10.3389/fpubh.2023.1089685)
Supplement: Supplementary file 5 [file Data_Sheet_5.PDF]

ID:

UNIVERSITÄT ZU LÜBECK
INSTITUT FÜR SOZIALMEDIZIN
UND EPIDEMIOLOGIE

Research project "Implementation and evaluation of a multilingual information service on rehabilitation for children and adolescents with a migration background (MiMi-Reha-Kids)"

Declaration of consent

Mr./Mrs. ____
First name; Last name (Please complete in block capitals)

Name of the child ____
First name; Last name (Please complete in block capitals)

I have been informed about the content and purpose of the research project. The research project is conducted under the responsibility of Prof. Dr. Bethge from the University of Lübeck. I have received the study information for the research project "Implementation and evaluation of a multilingual information offer for rehabilitation for children and adolescents with migration background (MiMi-Reha-Kids)". I have read and understood it.

I was assured that

1. participation is voluntary for myself and my child,
2. participation or non-participation will not affect any present or future benefits for myself or my child,
3. I can revoke my participation or the participation of my child at any time without disadvantages,
4. no personal details or other details that allow conclusions to be drawn about my person or my child will be passed on to third parties,
5. collected data (consent form, questionnaire data, routine data) are deleted no later than ten years after the end of the project.

I agree that routine data from my insurance account with German Pension Insurance (e.g. age of child, gender of child, approval diagnosis, start period of your child's rehab) will be merged with the survey data from the parent and child questionnaires, as described in the study information. I have been informed about my data protection rights. I agree to the collection, processing and storage of my data, as well as the transmission within the scope of the study.

I would like to support the research project and therefore agree to participate.

Place, date: ______________________________ Signature: ____________________________________
Insured person
